# Association Between Novel Lipid and Anthropometric Indices and Sleep Duration and Disturbance: A Cross-Sectional NHANES Study 2005–2020

**DOI:** 10.1155/ije/9976711

**Published:** 2025-10-30

**Authors:** Wangchen Yu, Emily Schembri, Alan C. Young, Denise M. O'Driscoll

**Affiliations:** ^1^Eastern Health Clinical School, Monash University, Victoria, Australia; ^2^Department of Respiratory and Sleep Medicine, Eastern Health, Victoria, Australia

**Keywords:** anthropometric indices, cardiometabolic risk, NHANES, novel lipid indices, sleep disturbance, sleep duration

## Abstract

**Objectives:**

Insufficient or excessive sleep and dyslipidemia are significant cardiovascular risk factors. Whilst the relationship between sleep duration and traditional lipid indices are well described, the connection to novel lipid and anthropometric indices remains unclear. This study examines these associations using National Health and Nutrition Examination Survey (NHANES) data from 2005 to 2020.

**Methods:**

This cross-sectional study analyzed data from 9847 adults from NHANES 2005–2020, excluding those with major cardiovascular disease and cancer. Sleep duration was categorized as insufficient (< 7 h), normal (7-8 h), and excessive (> 8 h). Self-reported sleep disturbance was documented. Novel indices included non-high-density lipoprotein cholesterol to high-density lipoprotein cholesterol ratio (NHHR), Triglyceride to HDL-Cholesterol (TG/HDL), Triglyceride-Glucose (TyG) Index, Visceral Adiposity Index (VAI), Lipid Accumulation Product (LAP), Conicity Index (CI), Body-Roundness Index (BRI), A Body Shape Index (ABSI), and Weight-adjusted waist index (WWI). Generalized additive models (GAMs) with spline smoothing and threshold analysis assessed nonlinear associations, adjusting for confounders. Weighted multivariate linear regression evaluated linear associations.

**Results:**

Insufficient sleep was associated with higher TyG combined with waist-to-height ratio (TyG–WHtR) (*p* = 0.003). Excessive sleep was linked to higher TyG–WHtR, CI, BRI, ABSI, and WWI (*p* < 0.001). Sleep disturbance was associated with elevated TyG–WHtR, TyG–WC, LAP, CI, BRI, ABSI, and WWI (*p* < 0.001). Threshold analysis confirmed significant changes in several indices, emphasizing the impact of both insufficient and excessive sleep.

**Conclusions:**

Insufficient, excessive sleep duration and sleep disturbance are associated with adverse lipid and anthropometric profiles, indicating increased cardiometabolic risk. Optimal sleep duration and addressing sleep disturbance could mitigate these risks. Further research is needed to understand the underlying mechanisms.

## 1. Introduction

Sleep disorders include conditions affecting sleep patterns, such as difficulties with sleep onset and maintenance, sleep-disordered breathing, and sleep-related movement disorders. Research into the effects of sleep on health has expanded rapidly. Insufficient and excessive sleep have both been linked to increased cardiovascular and all-cause mortality with current recommendations that adults achieve a minimum of 7 h sleep per night to maintain optimal health [1]. Dyslipidemia, which comprises the abnormal level of lipids in the blood, is a major risk factor for cardiovascular diseases [2]. Sleep loss and disturbances could potentially lead to cardiovascular disease via their effects on lipid profiles and anthropometric indices [3]. Disrupted sleep patterns may result in dysregulation of lipid metabolism and increased body fatness, both of which are established risk factors for cardiovascular disease.

Traditional lipid indices such as high-density lipoprotein (HDL), low-density lipoprotein (LDL), total cholesterol, and triglycerides (TGs) are commonly used to assess for the presence of dyslipidemia. Studies suggest that both insufficient and excessive sleep durations may be linked to abnormal lipid profiles, obesity, and atherogenic dyslipidemia across different ethnicity groups [4–6]. Particularly, short sleep duration is associated with low HDL cholesterol and high TG levels [7], while sleep disturbances are linked to abnormal TG and blood glucose levels [8]. Interestingly, long sleep duration has also been associated with low HDL-C levels and a poorer lipid profile over time [9]. Other short-term studies reported reductions in TGs and LDL-C with sleep restriction, suggesting possible beneficial effects on lipid profiles under specific conditions [10]. However, randomized controlled trials have shown no changes in lipid profiles after five nights of restricted sleep (< 4 h/night), highlighting the complexity of the relationship between sleep duration and lipid profiles [11].

Introducing novel lipid and anthropometric indices, such as the non-high-density lipoprotein cholesterol to high-density lipoprotein cholesterol ratio (NHHR), Triglyceride-Glucose (TyG) Index, Triglyceride to HDL-Cholesterol (TG/HDL-C) ratio, Visceral Adiposity Index (VAI), Lipid Accumulation Product (LAP), Conicity Index (CI), Body-Roundness Index (BRI), A Body Shape Index (ABSI), and Weight-Adjusted Waist Index (WWI), could clarify underlying mechanisms behind sleep duration and disturbances and lipid profile abnormalities. NHHR, a comprehensive indicator of atherosclerotic lipids, has shown superior predictive and diagnostic efficacy compared with traditional lipid parameters for conditions like metabolic syndrome, insulin resistance, diabetes, and obstructive sleep apnea (OSA) [12, 13]. Both TgG and TG/HDL-C ratio are significantly associated with insulin resistance and sleep disorders, including OSA, insomnia, and restless legs syndrome [14]. Elevated VAI and LAP levels are linked to a higher risk of OSA, with both also being practical markers for assessing the risk of metabolic disorders related to obesity and abdominal lipid accumulation [15, 16]. CI, based on waist circumference, body weight, and height, is positively associated with insulin resistance, hypertension, and dyslipidemia [17]. ABSI and BRI are linked to hypertriglyceridemia and high fasting blood sugar, though their predictive value for detecting dyslipidemia and cardiometabolic risk remains unclear [18, 19].

Given this background, we aimed to assess how sleep duration and sleep disturbances relate to novel lipid and anthropometric indices, in the adult population utilizing National Health and Nutrition Examination Survey (NHANES) data from 2005 to 2020. NHANES provides an exceptional opportunity to explore associations within a large, diverse sample of the adult population, as it collects comprehensive health, nutritional, and demographic data. This wealth of information allows for the adjustment of various potential confounders when examining the relationships between sleep characteristics and lipid profiles. By understanding these relationships, we can better identify potential targets for intervention to reduce the risk of dyslipidemia and its associated health complications.

## 2. Methods

Data from NHANES covering the years 2005–2020, prior to the COVID-19 pandemic, were analyzed. The study protocol was approved by the research ethics review board of the National Center for Health Statistics (NCHS) at the Centers for Disease Control and Prevention (CDC), and all participants provided written informed consent. Further details about NHANES can be found online (https://www.cdc.gov/nchs/nhanes/index.htm).

**Figure 1:**
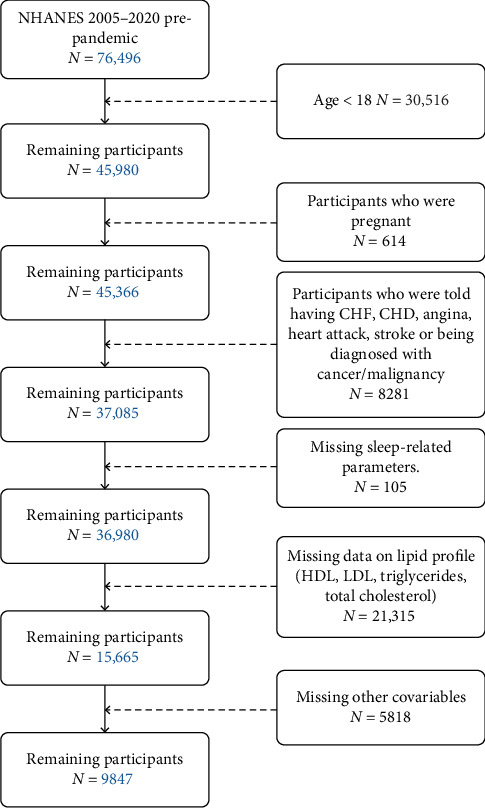
The flowchart depicting sample selection for the National Health and Nutrition Examination Survey (NHANES) from 2005 to 2020 prepandemic. CHF: congestive heart failure; CHD: coronary heart disease.

The total prepandemic sample size for NHANES 2005–2020 was 76,496 participants. The following exclusion criteria were applied to participants: (1) under 18 years of age; (2) pregnant; (3) medical history of congestive heart failure, coronary heart disease, angina, stroke, or cancer; (4) missing data on sleep-related parameters or lipid profiles; and (5) missing data on covariates such as education level, income–poverty ratio, hypertension, diabetes, body mass index, smoking status, and alcohol consumption (see Figure 1).

### 2.1. Sleep Duration and Disturbance

In accordance with earlier studies [20], sleep duration was categorized as insufficient (< 7 h/night), normal (7-8 h/night), or excessive (> 8 h/night). Sleep disturbance was defined as whether participants ever told a doctor or other health professional that they had trouble sleeping (yes vs. no). Participants who responded “do not know” or “refused” were considered as having missing responses.

### 2.2. Lipid and Anthropometric Indices

After a home interview, participants were examined at a mobile examination center (MEC) following a 9-h fast. The MEC measured height and weight according to a standardized protocol, calculating body mass index (BMI) to the nearest 0.1 cm.

Waist circumference was measured using an elastic tape positioned between the upper edge of the iliac crest and the lower edge of the 12th rib. The measurement was taken horizontally at the midpoint between these two landmarks. The waist-to-height ratio (WHtR) was determined by dividing waist circumference (m) by height (m). NHHR was calculated as non-HDL-C (mg/dL) divided by HDL-C (mg/dL), with non-HDL-C derived by subtracting HDL-C from total cholesterol (TC) (mg/dL). The TG/HDL ratio was computed by dividing TGs (mg/dL) by HDL-C (mg/dL).

Several indices related to TyG Index were calculated, combining BMI, WC, and WHtR. LAP, VAI, WWI, CI, BRI, and ABSI were further calculated using the following formulas [17, 21].

(1) $$\begin{array}{c} \text{TyG}=\log\;\left(\text{TG }\left(\text{mg}/\text{dL}\right)\times \text{fasting plasma glucose }\left(\text{mg}/\text{dL}\right)/2\right),\\ \text{TyG}-\text{BMI}=\text{TyG}\times \text{BMI }\left(\text{kg}/m^{2}\right),\\ \text{TyG}-\text{WC}=\text{TyG}\times \text{WC }\left(m\right),\\ \text{TyG}-\text{WHtR}=\text{TyG}\times \text{WHtR},\\ \text{LAP }\left(\text{Male}\right)=\left(\text{WC }\left(\text{cm}\right)-65\right)\times \text{TG }\left(\text{mmol}/L\right),\\ \text{LAP }\left(\text{Female}\right)=\left(\text{WC }\left(\text{cm}\right)-58\right)\times \text{TG}\left(\text{mmol}/L\right),\\ \text{VAI }\left(\text{Male}\right)=\left(\frac{\text{WC }\left(\text{cm}\right)}{39.68+1.88\times \text{BMI}}\right)\times \left(\frac{\text{TG}}{1.03}\right)\times \left(\frac{1.31}{\text{HDL}\left(\text{mmol}/L\right)}\right),\\ \text{VAI }\left(\text{Female}\right)=\left(\frac{\text{WC}\left(\text{cm}\right)}{36.58+1.89\times \text{BMI}}\right)\times \left(\frac{\text{TG}}{0.81}\right)\times \left(\frac{1.52}{\text{HDL}\left(\text{mmol}/L\right)}\right),\\ \text{WWI}=\frac{\text{WC }\left(m\right)}{{\text{weight }\left(\text{kg}\right)}^{2}},\\ \text{CI}=\frac{\text{WC }\left(m\right)}{0.109\times \sqrt{\left(\text{weight }\left(\text{kg}\right)/\text{height }\left(m\right)\right)}},\\ \text{BRI}=364.2-365.5\times \sqrt{1-{\left(\frac{\text{WC }\left(m\right)/\left(2\pi \right)}{0.5\times \text{height }\left(m\right)}\right)}^{2}},\\ \text{ABSI}=\frac{\text{WC}\left(m\right)}{{\text{height}\left(m\right)}^{\left(1/2\right)}\times {\text{BMI}\left(\text{kg}/m^{2}\right)}^{\left(2/3\right)}}. \end{array}$$

### 2.3. Covariates

The final analysis adjusted for potential confounding factors related to sleep and cardiometabolic risk, including demographic variables such as age, race, gender, educational level, marital status, and the income-to-poverty ratio (PIR). Races were divided into Mexican Americans, non-Hispanic whites, non-Hispanic blacks, Hispanics, and other races and education levels were divided into less than high school, high school, and more than high school. Smokers were defined as never, former, or current smokers, while hypertension was defined as mean systolic blood pressure ≥ 140 mmHg or mean diastolic blood pressure ≥ 90 mmHg. Diabetes was defined as a self-reported physician diagnosis of diabetes, categorized into diabetic, nondiabetic, and borderline. Alcohol consumption was categorized as drinker (> 12 times a year) and nondrinker.

### 2.4. Statistical Analysis

To ensure the sample's representativeness within the population, the analysis was weighted using appropriate sample weights as detailed on the NHANES website. The data from 2017 to March 2020 cover a 3.2-year period, compared with the standard 2-year period of other NHANES cycles. Weights for the combined NHANES survey cycles were adjusted and calculated in accordance with NHANES guidelines (NHANES, 2017–March 2020 Prepandemic File: Sample Design, Estimation, and Analytic Guidelines, accessible at https://wwwn.cdc.gov/nchs/nhanes/analyticguidelines.aspx).

All data analyses were conducted using R software (Version 4.3.2), STATA (Version 18), and EmpowerStat (https://www.empowerstats.net/cn/). We present the baseline characteristics as weighted means with 95% confidence intervals (CIs) for continuous variables and as weighted percentages with 95% CIs for categorical variables.

Considering the inconsistent associations between sleep duration and various lipid groups, as well as the U-shaped relationship between sleep and cardiometabolic outcomes reported in prior studies [7, 22, 23], a nonlinear relationship between sleep and our indices was expected. Thus, we modeled sleep duration with survey-weighted smoothing spline curves within generalized additive models (GAM). GAMs extend generalized linear models by accommodating nonlinear relationships between predictors and outcomes. Model assumptions were assessed by checking the residuals for normality, symmetry, and homoscedasticity.

In the GAM analysis, the novel lipid and anthropometric indices were treated as outcomes, and their adjusted relationships to sleep duration were modeled with smooth functions under survey weights. The adjusted models included confounders such as age, gender, race, marital status, PIR, BMI, hypertension, diabetes, smoking status, and alcohol consumption. The effective degree of freedom (EDF) in GAM represents the complexity of the smooth, where an EDF of 1 indicates a linear association, and values greater than 1 suggest a more intricate relationship between lipid profiles and sleep duration. Plots of the estimated smooth functions, accompanied by 95% CIs, were produced for the adjusted GAMs. To summarize nonlinear patterns and identify thresholds, we additionally fit survey-weighted two-piecewise (segmented) linear regression models. Inflection point(s) (knot[s]) were selected by profile likelihood, and nonlinearity was evaluated with a likelihood-ratio test (LRT) comparing the two-piece model to a single-slope linear model. When LRT *p* < 0.05, we reported the inflection point(s) and segment slopes with 95% CIs; otherwise, a single linear association was deemed adequate.

In the instances of an EDF of, or close to 1, we explored linear associations using univariate and multivariable survey-weighted linear regression. Model I was unadjusted, while Model II included adjustments for age, gender, education level, race, marital status, and PIR. Model III further adjusted for BMI, diabetes status, hypertension, smoking status, and alcohol intake. In the main manuscript we present Model III; Models I–II are provided in the Supporting Information. A *p* value below 0.05 was deemed statistically significant.

## 3. Results

The final sample included 9847 participants, representing 59,367,915 US adults aged 18 and older. Baseline characteristics are summarized in Table 1 by sleep duration groups and sleep disturbance groups. Participants with insufficient sleep were more likely to be male, younger, and have lower education levels, with 15.57% having less than a high school education. This group also had a higher prevalence of non-Hispanic Black and Mexican American individuals. The normal sleep group had a balanced gender distribution, a slightly older mean age, and the highest percentage of non-Hispanic Whites, with 67.69% having more than a high school education and a higher mean PIR, indicating better socioeconomic status. The excessive sleep group had a higher proportion of females, a similar age distribution to the normal sleep group, a higher prevalence of non-Hispanic Whites, and a moderate educational distribution.

Participants with sleep disturbance were more likely to be female, older (mean age 47.3 years), and unmarried, with higher rates of obesity, higher mean weight, and a greater prevalence of hypertension and diabetes. Conversely, those without sleep disturbance were more likely to be male, younger (mean age 43.4 years), married, and had lower rates of obesity and diabetes. Significant differences were observed in gender, race, education level, marital status, PIR, BMI, weight, WHtR, WC, history of hypertension and diabetes, smoking status, and alcohol intake among the three sleep duration categories. Similar significance was seen between the two sleep disturbance groups, with age being significant while education level, PIR, and history of hypertension were not (*p* > 0.05).

### 3.1. Lipid and Anthropometric Indices According to Sleep Duration and Sleep Disturbance

Table 2 shows the weighted distribution of lipid and anthropometric indices by sleep duration groups and sleep disturbance groups, respectively. HDL levels are significantly lower in individuals with insufficient sleep (*p* < 0.001). While TG levels do not show a significant difference across sleep duration groups, they are notably higher in those with sleep disturbance (*p* < 0.001). The TG/HDL ratio is significantly elevated in individuals with insufficient sleep (*p* = 0.027) and those experiencing sleep disturbance (*p* = 0.004). NHHR is significantly higher in the insufficient sleep group but not among those with sleep disturbance. Conversely, the TyG Index shows significant differences between the sleep disturbance groups (*p* < 0.001) but not among different sleep duration categories. The TyG–WHtR, TyG–WC, and TyG–BMI indices are significantly higher in individuals with insufficient sleep (*p* < 0.001) and in those with sleep disturbance (*p* < 0.001). Additionally, anthropometric indices such as CI, BRI, ABSI, and WWI exhibit significant differences across sleep duration groups, with both insufficient and excessive sleep associated with higher values (*p* < 0.001). These indices are also significantly elevated in individuals with sleep disturbance (*p* < 0.001).

### 3.2. Lipid and Anthropometric Indices—GAM and Multivariate Linear Regression Model

**Figure 2:**
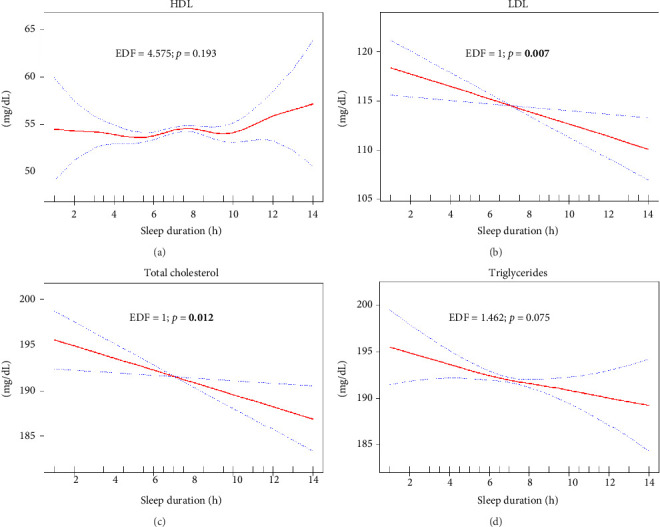
Plots of adjusted estimated smoothing spline function of sleep duration with 95% CI band for generalized additive model (GAM) when the response variables was (a) HDL (mg/dL); (b) LDL (mg/dL); (c) total cholesterol (mg/dL); and (d) triglycerides (mg/dL). EDF: effective degree of freedom. Significant *p* values are bolded.

**Figure 3:**
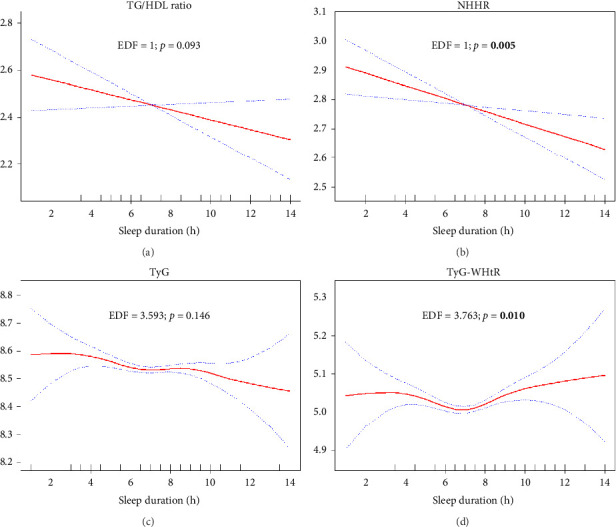
Plots of adjusted estimated smoothing spline function of sleep duration with 95% CI band for generalized additive model (GAM) when the response variables was (a) TG/HDL; (b) NHHR; (c) TyG; and (d) TyG–WHtR. EDF: effective degree of freedom. Significant *p* values are bolded.

**Figure 4:**
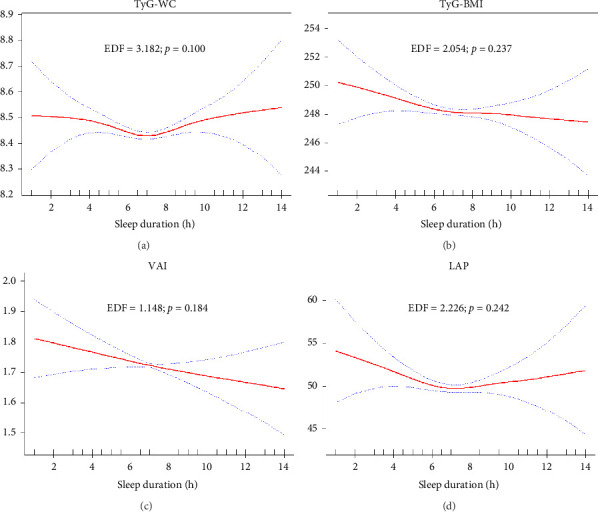
Plots of adjusted estimated smoothing spline function of sleep duration with 95% CI band for generalized additive model (GAM) when the response variables was (a) TyG–WC; (b) TyG–BMI; (c) VAI; and (d) LAP. EDF: effective degree of freedom. Significant *p* values are bolded.

**Figure 5:**
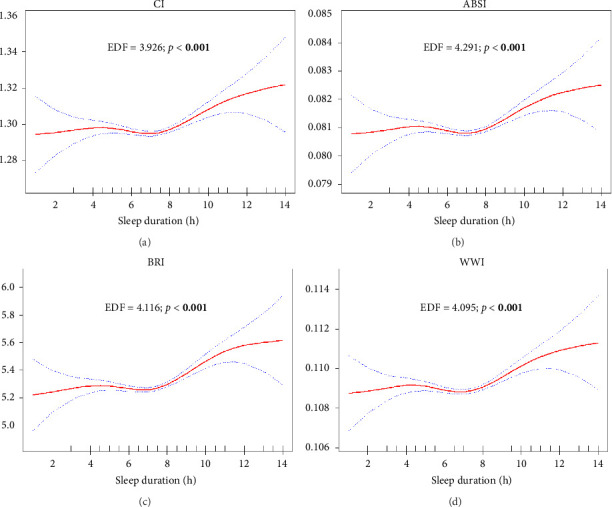
Plots of adjusted estimated smoothing spline function of sleep duration with 95% CI band for generalized additive model (GAM) when the response variables was (a) CI; (b) ABSI; (c) BRI; and (d) WWI. EDF: effective degree of freedom. Significant *p* values are bolded.

The analysis of the relationship between sleep duration and various lipid and anthropometric indices, using crude (Supporting Figures S1–S16) and survey-weighted smoothing spline curves (Figures 2, 3, 4, and 5) and two-piece (threshold) analysis (Table 3), revealed complex, often nonlinear associations. For HDL, significant changes were observed between 5.5 and 7.5 h of sleep (*p* = 0.025), although the GAM indicated an insignificant nonlinear association after adjusting for confounders (*p* = 0.193). Both TyG–WHtR and TyG–WC exhibited a significant inflection point at 6.5 h of sleep (*p* < 0.001 and *p* = 0.004), indicating a U-shaped relationship where sleep durations less than and more than 6.5 h were notably associated with higher levels of these indices. However, TyG–WC did not remain significant in GAM after adjusting for confounders (*p* = 0.100). While TyG and TyG–BMI showed nonlinear relationships in the smoothing curves, their *p* values were not significant (*p* = 0.250 and *p* = 0.067), suggesting a potential linear association. LAP showed a significant knot at 6.5 h with a negative slope below the knot (*p* = 0.011), suggesting that shorter sleep durations are strongly linked to higher LAP values, with the strength of this association diminishing as sleep duration increases. Similar findings were observed for CI, BRI, ABSI, and WWI, with two knots (CI/BRI/ABSI: 4.5 and 7.5 h; WWI: 4.0 and 6.5 h) and positive upper-segment slopes (all LRT *p* < 0.001), consistent with higher anthropometric indices at longer sleep durations. Complete segment coefficients and Wald *p* values are provided in Supporting Table S1. Indices such as LDL, total cholesterol, TGs, TG/HDL ratio, NHHR, and VAI exhibited adjusted EDFs close to or equal to 1, indicating linear relationships, which were analyzed using survey-weighted multivariable linear regression (Table 4; Model III). Significant results were found in crude or partially adjusted models for TGs, TG/HDL ratio, NHHR, and VAI, while changes in LDL and total cholesterol levels remained insignificant across different sleep duration categories. For clarity, only fully adjusted estimates (Model III; sleep duration categorical with Normal 7-8 h as reference) are presented in the main text; crude and partially adjusted results (Models I–II) are provided in Supporting Table S2.

The associations between lipid and anthropometric indices and sleep disturbance, detailed in Table 5, were analyzed using survey-weighted multivariable linear regression (Model III; exposure = Present vs. Absent [reference]). Traditional lipid indices such as HDL-C, LDL-C, and total cholesterol levels did not show significant differences between individuals with and without sleep disturbance. Mild significance was noted for TGs, TG/HDL ratio, NHHR, and TyG in partially adjusted or unadjusted models. Several anthropometric indices, including TyG–WHtR and TyG–WC, were significantly higher in individuals with sleep disturbance, indicating a compounded metabolic burden. LAP was also notably elevated, highlighting a higher risk of visceral fat accumulation and associated cardiometabolic risks among those with poor sleep quality. VAI and TyG–BMI showed significance only in unadjusted or partially adjusted models. Other anthropometric indices, such as CI, BRI, ABSI, and WWI, exhibited strong associations with sleep disturbance when confounders were considered, further underscoring the adverse impact of poor sleep on body composition and metabolic health. As above, only fully adjusted estimates (Model III) are shown in Table 5; crude and partially adjusted results (Models I–II) are moved to Supporting Table S3.

## 4. Discussion

This study presents the first thorough analysis of the association between novel lipid and anthropometric indices and sleep. TyG–WHtR was positively associated with both insufficient sleep (< 7 h/night) and excessive sleep (> 8 h/night). CI, BRI, ABSI, and WWI were positively associated with excessive sleep duration (> 8 h/night). Additionally, TyG–WHtR, TyG–WC, LAP, CI, BRI, ABSI, and WWI were positively associated with sleep disturbance.

### 4.1. Sleep Duration and Novel Lipid and Anthropometric Indices

We report an association between short sleep duration and low HDL levels in crude and adjusted linear regression models, which is in consistent with a recent study from the Fasa Adults Cohort Study [24]. It is worth noting that the assumption of linearity was then violated as GAM exhibits a nonlinearity trend for HDL. Threshold analysis confirmed a significant rise in HDL levels between 5.5 and 7.5 h of sleep, indicating a potential protective effect on HDL during the proposed sleep duration. The finding of nonlinearity roughly aligns with previous literature, where a smaller NHANES population was also analyzed using GAM [7]. However, this nonlinear relationship warrants further investigation, as HDL changes became insignificant after adjusting for confounders in GAM in our study. Sleep duration showed no association with LDL cholesterol, total cholesterol, and TGs levels, neither via GAM nor linear regression models. This finding supports previously published systematic review and meta-analyze that reported no significant associations between short or long sleep duration and the risk of serum lipid abnormality (high TG, high LDL-C, and low HDL-C), reinforcing the complexity of these relationships [25].

Regarding novel lipid indices, NHHR and TG/HDL exhibited partial linear associations with insufficient sleep duration, while TyG showed no clear associations with sleep changes, despite GAM suggesting nonlinearity. Thus, no firm conclusions can be drawn about their associations with sleep duration in our study.

As aforementioned, TyG–WHtR demonstrated significant changes in both insufficient and excessive sleep, with a symmetrical U-shape observed via spline smoothing, indicating a minimum value at 6.5 h of sleep. Similar curves were observed in TyG–BMI and TyG–WC, albeit with inconsistent significance in GAM. Being one of the strongest predictors of metabolic syndrome and atherosclerotic cardiovascular disease, as suggested in emerging literature [26], the association between TyG–WHtR and altered sleep duration may shed light on the potential negative effects of both insufficient and excessive sleep on metabolic health. These findings highlight the importance of maintaining optimal sleep duration to mitigate the risk of adverse cardiometabolic outcomes and underscore the need for further research to elucidate the mechanisms underlying these associations.

Recently proposed anthropometric and metabolic indices such as VAI and LAP showed distinctive results. The fitted GAM analysis indicated a fairly linear association between VAI and sleep duration, with no significant changes noted in multivariate linear regression. This contrasts with previous literature reporting a significant nonlinear (L-shaped) relationship, suggesting a negative association between short sleep and VAI levels using the NHANES data [23]. It is noted that slightly different NHANES cycles were extracted and survey sample weighting was not considered in previous literature, which may explain the differing results. For LAP, an inflection point at 6.5 h of sleep was observed, with significant changes for shorter sleep durations in threshold effect analysis, although GAM did not remain significant after considering all confounders. This finding generally aligns with a study showing a sex-specific association between short sleep duration and elevated LAP levels in a Chinese population [27]. However, no prior studies have reported a nonlinearity of LAP with sleep duration.

Newly introduced indices such as CI, BRI, ABSI, and WWI showed similar nonlinear patterns in GAM, with significant changes linked to excessive sleep durations. Although evidence has been limited, data from MASHAD cohort recently demonstrated lower ABSI and WWI among long sleepers and higher ABSI/WWI with short sleep, supporting that sleep duration can map onto these ‘shape indices in longitudinal data [28]. BRI has been demonstrated to be a superior indicator for predicting clustering of cardiometabolic abnormalities compared with BMI, WC, and ABSI in both men and women [29]. In older adults, BRI and WHtR were the most accurate indices for predicting high cardiometabolic risk factors [30]. CI was positively associated with insulin resistance, hypertension, and dyslipidemia in rural South African adults [17]. WWI was proposed as a new index to predict cardiometabolic morbidity and mortality, showing excellent predictive power, especially when combined with BMI [31]. Our findings highlight the potential impact of excessive sleep on cardiometabolic conditions, suggesting that the nonlinear associations observed with these novel indices could provide new insights into the complex relationship between sleep duration and cardiometabolic health.

The clear underlying mechanisms responsible for the association between sleep duration, lipid abnormalities anthropometric changes as well as cardiometabolic risks remain to be elucidated. However, there is evidence that sleep deprivation alters the secretion of hormones such as cortisol, growth hormone, and ghrelin. Increased cortisol levels contribute to glucose intolerance and central adiposity, while altered ghrelin levels affect appetite regulation, promoting obesity [32]. Furthermore, sleep insufficiency may also affect adipokine levels, such as increased leptin and decreased adiponectin, contributing to obesity and insulin resistance [11]. Importantly, the association may be bidirectional: higher adiposity can also disrupt sleep; in Iranian adults, overweight/obesity was linked to greater odds of nocturnal sleep disruption [33]. Inflammation is a key biological pathway, with both short and long sleep durations increasing inflammatory markers like C-reactive protein (CRP) and interleukin-6 (IL-6) [8]. Lastly, insufficient sleep may impair endothelial function, reducing the production of nitric oxide which is critical for vasodilation [34]. This dysfunction can lead to hypertension and increased cardiovascular risk. Regardless of the underlying cause, short duration of sleep has found to be associated with increased morbidity and mortality [35].

### 4.2. Sleep Disturbance and Novel Lipid and Anthropometric Indices

Significant associations between self-reported sleep disturbance and various lipid and anthropometric indices have been reported, underscoring the importance of sleep quality in metabolic health have been reported [8]. Although the presence of sleep disorders such as OSA and insomnia could not be directly assessed in our study as they were not asked about over the entire data collection time frame, fragmented and nonrestorative sleep disturbance are usually reported in patients suffering with these sleep disorders [36]. Previous research has linked OSA with high levels of total cholesterol, TGs, LDL-C, ApoB, and low levels of HDL-C, identifying LDL-C as an independent risk factor for OSA [37]. Our findings support this to some extent, as changes in TGs were significantly related to sleep disturbance. Participants with sleep disturbance exhibited elevated TG/HDL ratios and NHHR, although these associations lost significance after adjusting for confounders. The elevated TG/HDL ratio suggests a potential increased atherogenic risk, indicating higher cardiovascular disease risk among individuals with disrupted sleep [14]. NHHR has been reported in a very recent study using a smaller population from the NHANES database, as being a potential tool for OSA prediction [13]. Its risk predictive value has shown to be slightly superior than the traditional lipid parameters (HDL and TC). However, the relationship between NHHR and sleep disturbance remains unclear, as our study did not find a firm association, noting OSA was not a direct outcome.

Additionally, the TyG Index, which combines fasting glucose and TG levels, was higher in individuals with sleep disturbance but lost significance in the fully adjusted model. This index is a proposed marker for insulin resistance, implying that poor sleep quality may contribute to metabolic dysregulation and potential progression to type 2 diabetes [38]. Previous studies using NHANES data reported a positive association between TyG levels and increased OSA risk [14], and recent systemic reviews and meta-analyses have further highlighted the value of TyG in diagnosing and evaluating OSA prognosis [39, 40]. The significantly higher TyG–WHtR and TyG–WC indices in our study reinforce the notion of a compounded metabolic burden and increased cardiometabolic risk.

Both VAI and LAP showed some association with the presence of sleep disturbance, with LAP exhibiting significant changes in the fully adjusted model, indicating a link between poor sleep quality and visceral fat accumulation. This is again, consistent with previous studies supporting the use of LAP in clinical practice when evaluating cardiometabolic risk in patients with OSA [41]. Other anthropometric indices such as CI, BRI, ABSI, and WWI were significantly elevated in participants with sleep disturbance, indicating central adiposity and overall body fat distribution, critical risk factors for metabolic syndrome, and cardiovascular diseases. Previous studies have reported the superior prognostic value of CI than BMI for cardiovascular events in OSA patients [42], and the correlation between BRI or body composition changes and OSA, with significant associations between BRI and cardiovascular risk in hypertensive patients with OSA [43]. Although specific studies directly linking WWI to sleep disturbance or OSA were not identified, WWI has shown strong predictive performance for cardiometabolic risks, which are often associated with sleep disturbance [31].

### 4.3. Strengths and Limitations

The robustness of this study is underscored by its use of a large and geographically diverse sample of US adults from NHANES. Comprehensive adjustments for covariates and the application of GAM to uncover the nonlinear association between sleep and lipid and anthropometric indices further strengthen the findings. However, several limitations must be noted. First, the reliance on self-reported sleep disturbance and durations introduces the possibility of recall bias. Using more precise measures of sleep-related variables, such as polysomnography, could reduce variability, though such data are not available in the NHANES database. This issue has also been observed in prior NHANES studies, where the accuracy of self-reported sleep problems remains questionable [8]. Second, the cross-sectional design of the study limits its ability to infer causal relationships between sleep problems and lipid and anthropometric indices. Third, due to data limitations within NHANES, specific sleep disorders such as OSA and insomnia could not be precisely defined. Consequently, the relationships between sleep disturbance identified in this study were interpreted with caution, particularly when considering the clinical implications of specific sleep-related conditions.

## 5. Conclusion

We present a robust, large-sample analysis of novel lipid and anthropometric indices in relation to sleep patterns, leveraging the representativeness of NHANES across multiple survey cycles. We specifically revealed significant associations between TyG–WHtR and sleep insufficiency, as well as between CI, BRI, ABSI, and WWI with excessive sleep duration. TyG–WHtR, TyG–WC, LAP, CI, BRI, ABSI, and WWI all tend to be strongly associated with the presence of sleep disturbance. Future research should focus on sleep-related interventions to reduce the risk of dyslipidemia and its associated health complications.

## Figures and Tables

**Table 1:** Characteristics of the study population by sleep duration and sleep disturbances groups, survey-weighted.

Characteristics	Total (*n* = 9847)	Sleep duration			*p* value	Sleep disturbances		*p* value
		Insufficient (< 7 h) (*n* = 3341)	Normal (7-8 h) (*n* = 5105)	Excessive (> 8 h) (*n* = 1401)		Absent (*n* = 7501)	Present (*n* = 2346)	
Gender					**< 0.001**			**< 0.001**
Male	5197 (52.8%)	1825 (54.6%)	2738 (53.6%)	634 (45.3%)		4145 (55.3%)	1052 (44.8%)	
Female	4650 (47.2%)	1516 (45.4%)	2367 (46.4%)	767 (54.7%)		3356 (44.7%)	1294 (55.2%)	
Age, years (mean ± SD)	46.02 ± 16.32	45.46 ± 15.12	46.14 ± 16.47	46.92 ± 18.32	0.015	45.16 ± 16.51	48.79 ± 15.34	**< 0.001**
Race					**< 0.001**			**< 0.001**
Mexican American	1523 (15.5%)	492 (14.7%)	838 (16.4%)	193 (13.8%)		1281 (17.1%)	242 (10.3%)	
Non-Hispanic Black	2079 (21.1%)	963 (28.8%)	821 (16.1%)	295 (21.1%)		1610 (21.5%)	469 (20.0%)	
Non-Hispanic White	4161 (42.3%)	1211 (36.2%)	2377 (46.6%)	573 (40.9%)		2955 (39.4%)	1206 (51.4%)	
Other Hispanic	982 (10.0%)	349 (10.4%)	491 (9.6%)	142 (10.1%)		766 (10.2%)	216 (9.2%)	
Other race—including multiracial	1102 (11.2%)	326 (9.8%)	578 (11.3%)	198 (14.1%)		889 (11.9%)	213 (9.1%)	
Education					**< 0.001**			0.245
Less than high school	2050 (20.8%)	728 (21.8%)	1021 (20.0%)	301 (21.5%)		1620 (21.6%)	430 (18.3%)	
Completed high school	2236 (22.7%)	804 (24.1%)	1071 (21.0%)	361 (25.8%)		1715 (22.9%)	521 (22.2%)	
More than high school	5561 (56.5%)	1809 (54.1%)	3013 (59.0%)	739 (52.7%)		4166 (55.5%)	1395 (59.5%)	
Marriage					**< 0.001**			**< 0.001**
Married	5252 (53.3%)	1692 (50.6%)	2853 (55.9%)	707 (50.5%)		4108 (54.8%)	1144 (48.8%)	
Unmarried	4595 (46.7%)	1649 (49.4%)	2252 (44.1%)	694 (49.5%)		3393 (45.2%)	1202 (51.2%)	
PIR (mean ± SD)	2.63 ± 1.64	2.54 ± 1.61	2.76 ± 1.65	2.35 ± 1.61	**< 0.001**	2.63 ± 1.63	2.63 ± 1.68	0.953
BMI, kg/m^2^ (mean ± SD)	28.94 ± 6.73	29.53 ± 7.00	28.51 ± 6.38	29.11 ± 7.22	**< 0.001**	28.54 ± 6.45	30.23 ± 7.43	**< 0.001**
Hypertension					**0.009**			0.288
Hypertensive	1610 (16.4%)	573 (17.2%)	781 (15.3%)	256 (18.3%)		1210 (16.1%)	400 (17.1%)	
Nonhypertensive	8237 (83.6%)	2768 (82.8%)	4324 (84.7%)	1145 (81.7%)		6291 (83.9%)	1946 (82.9%)	
Smoking status					**< 0.001**			**< 0.001**
Current smoker	2215 (22.5%)	911 (27.3%)	960 (18.8%)	344 (24.6%)		1572 (21.0%)	643 (27.4%)	
Former smoker	2367 (24.0%)	722 (21.6%)	1322 (25.9%)	323 (23.1%)		1717 (22.9%)	650 (27.7%)	
Never-smoker	5265 (53.5%)	1708 (51.1%)	2823 (55.3%)	734 (52.4%)		4212 (56.2%)	1053 (44.9%)	
Alcohol intake					**< 0.001**			**0.002**
Drinker	1360 (13.8%)	396 (11.9%)	648 (12.7%)	316 (22.6%)		991 (13.2%)	369 (15.7%)	
Nondrinker	8487 (86.2%)	2945 (88.1%)	4457 (87.3%)	1085 (77.4%)		6510 (86.8%)	1977 (84.3%)	
Diabetes (%)					**< 0.001**			**< 0.001**
Borderline	187 (1.9%)	70 (2.1%)	95 (1.9%)	22 (1.6%)		127 (1.7%)	60 (2.6%)	
Diabetic	936 (9.5%)	327 (9.8%)	434 (8.5%)	175 (12.5%)		614 (8.2%)	322 (13.7%)	
Nondiabetic	8724 (88.6%)	2944 (88.1%)	4576 (89.6%)	1204 (85.9%)		6760 (90.1%)	1964 (83.7%)	

*Note:* Continuous variables are presented as the mean ± standard deviation (SD) using survey weights. Categorical variables are presented as number (unweighted) and percentage (survey weighted). *p* values are from survey-weighted linear regression (continuous variables) and survey-weighted Chi-square tests (categorical variables). The bold values indicate significant *p* values (*p* < 0.05).

Abbreviations: BMI, body mass index; PIR, poverty-income ratio.

**Table 2:** Lipid and anthropometric indices by sleep duration and sleep disturbances groups, weighted.

Index (mean ± SD)	Total (*n* = 9847)	Sleep duration			*p* value	Sleep disturbances		*p* value
		Insufficient (< 7 h) (*n* = 3341)	Normal (7-8 h) (*n* = 5105)	Excessive (> 8 h) (*n* = 1401)		Absent (*n* = 7501)	Present (*n* = 2346)	
HDL	54.21 ± 15.96	53.09 ± 15.72	54.81 ± 16.03	54.67 ± 16.16	**< 0.001**	54.20 ± 15.74	54.23 ± 16.66	0.947
LDL	114.52 ± 35.03	115.27 ± 34.93	114.76 ± 34.64	111.86 ± 36.53	0.007	114.68 ± 34.58	114.00 ± 36.42	0.411
Triglycerides	114.05 ± 65.39	115.43 ± 65.95	114.02 ± 64.75	110.85 ± 66.31	0.089	112.11 ± 64.71	120.26 ± 67.17	**< 0.001**
Total cholesterol	191.54 ± 39.76	191.45 ± 39.62	192.38 ± 39.45	188.70 ± 41.08	**0.009**	191.31 ± 39.23	192.29 ± 41.41	0.299
TG/HDL ratio	2.45 ± 2.00	2.53 ± 2.03	2.42 ± 1.93	2.39 ± 2.18	**0.028**	2.40 ± 1.94	2.60 ± 2.19	**< 0.001**
NHHR	2.78 ± 1.25	2.86 ± 1.29	2.75 ± 1.19	2.70 ± 1.35	**< 0.001**	2.77 ± 1.21	2.81 ± 1.37	0.245
TyG	8.54 ± 0.63	8.55 (8.51,8.58)	8.54 ± 0.62	8.52 ± 0.66	0.245	8.51 ± 0.62	8.62 ± 0.63	**< 0.001**
TyG–WHtR	5.02 ± 1.01	5.08 ± 1.02	4.97 ± 0.98	5.10 ± 1.10	**< 0.001**	4.95 ± 0.98	5.26 ± 1.07	**< 0.001**
TyG–WC	8.45 ± 1.71	8.56 ± 1.74	8.36 ± 1.65	8.49 ± 1.82	**< 0.001**	8.33 ± 1.65	8.82 ± 1.83	**< 0.001**
TyG–BMI	248.25 ± 64.84	253.56 ± 66.68	244.48 ± 62.02	249.31 ± 69.42	**< 0.001**	244.07 ± 62.13	261.62 ± 71.23	**< 0.001**
VAI	1.72 ± 1.38	1.76 ± 1.38	1.69 ± 1.34	1.74 ± 1.52	0.078	1.67 ± 1.32	1.90 ± 1.53	**< 0.001**
LAP	50.11 ± 40.23	51.74 ± 41.03	48.89 ± 38.96	50.67 ± 42.70	**0.005**	47.65 ± 38.47	57.98 ± 44.51	**< 0.001**
CI	1.30 ± 0.09	1.30 ± 0.09	1.29 ± 0.09	1.31 ± 0.10	**< 0.001**	1.29 ± 0.09	1.32 ± 0.09	**< 0.001**
BRI	5.29 ± 2.29	5.42 ± 2.35	5.14 ± 2.16	5.54 ± 2.56	**< 0.001**	5.13 ± 2.20	5.81 ± 2.50	**< 0.001**
ABSI	0.08 ± 0.00	0.08 ± 0.00	0.08 ± 0.00	0.08 ± 0.01	**< 0.001**	0.08 ± 0.00	0.08 ± 0.00	**< 0.001**
WWI	0.11 ± 0.01	0.11 ± 0.01	0.11 ± 0.01	0.11 ± 0.01	**< 0.001**	0.11 ± 0.01	0.11 ± 0.01	**< 0.001**

*Note:* Values are survey-weighted means ± SD. *p* values from survey-weighted linear regression comparing groups. Lipids in mg/dL; indices dimensionless. TyG–WHtR: TyG related to WHtR; TyG–WC: TyG related to WC; TyG–BMI: TyG related to BMI. NHHR: non-high-density lipoprotein cholesterol to high-density lipoprotein cholesterol ratio. The bold values indicate significant *p* values (*p* < 0.05).

Abbreviations: ABSI, A Body Shape Index; BRI, Body-Roundness Index; CI, Conicity Index; HDL, high-density lipoprotein; LAP, lipid accumulation product; LDL, low-density lipoprotein; non-HDL-C, non-high-density lipoprotein cholesterol; TG/HDL ratio, total cholesterol to high-density lipoprotein ratio; TyG, Triglyceride-Glucose Index; VAI, Visceral Adiposity Index; WWI, Weight-Adjusted Waist Index.

**Table 3:** Threshold effect analysis of sleep duration on lipid and anthropometric indices using a survey-weighted two-piecewise linear regression model.

Outcome	Inflection point(s), (h)	Segment slopes β (95% CI) per 1 h change		*p* for non-linearity (LRT)
HDL	5.5, 7.5	< 5.5	−0.44 (−1.23, 0.35)	**0.025**
		5.5–7.5	**0.82 (0.20, 1.45)**	
		≥ 7.5	0.02 (−0.52, 0.55)	

TyG	5.5	< 5.5	−0.023 (−0.052, 0.005)	0.250
		≥ 5.5	−0.004 (−0.0136, 0.0051)	

TyG–WHtR	6.5	< 6.5	**−0.62 (−1.13, −0.01)**	**< 0.001**
		≥ 6.5	0.08 (−0.27, 0.43)	

TyG–WC	6.5	< 6.5	−0.032 (−0.057, −0.008)	**0.004**
		≥ 6.5	**0.019 (0.003, 0.036)**	

TyG–BMI	6.5	< 6.5	**−0.617 (−1.134, −0.010)**	0.067
		≥ 6.5	0.080 (−0.273, 0.434)	

LAP	6.5	< 6.5	**−1.326 (−2.301, −0.351)**	**0.011**
		≥ 6.5	0.502 (−0.165, 1.169)	

CI	4.5, 7.5	< 4.5	0.003 (−0.004, 0.009)	**< 0.001**
		4.5–7.5	−0.002 (−0.004, 0.000)	
		≥ 7.5	**0.006 (0.004, 0.008)**	

BRI	4.5, 7.5	< 4.5	0.027 (−0.046, 0.099)	**< 0.001**
		4.5–7.5	−0.014 (−0.037, 0.009)	
		≥ 7.5	**0.082 (0.054, 0.109)**	

WWI	4, 6.5	< 4	0.0003 (−0.0003, 0.0009)	**< 0.001**
		4–6.5	−0.0003 (−0.0006, 0.0001)	
		≥ 6.5	**0.0004 (0.0003, 0.0006)**	

ABSI	4.5, 7.5	< 4.5	0.0002 (−0.0002, 0.0006)	**< 0.001**
		4.5–7.5	−0.0001 (−0.0002, 0.0000)	
		≥ 7.5	**0.0004 (0.0002, 0.0005)**	

*Note:* Survey-weighted models adjusted for sex, age, education, race/ethnicity, marital status, poverty-income ratio, BMI, diabetes, hypertension, smoking, and alcohol use. *p* for nonlinearity (LRT) compares a two-piecewise model to a single-slope linear model. Bold β indicates 95% CI excluding 0. Significant *p* for nonlinearity (LRT) and segment slopes whose 95% CI excludes 0 are bolded.

Abbreviations: ABSI, A Body Shape Index; BMI, body mass index; BRI, Body-Roundness Index; CI, Conicity Index; HDL, high-density lipoprotein; LAP, lipid accumulation product; TyG, Triglyceride-Glucose Index; WC, waist circumference; WHtR, waist-to-height ratio; WWI, Weight-Adjusted Waist Index.

**Table 4:** Survey-weighted multivariable linear regression of lipid/anthropometric indices by sleep-duration category (reference = 7-8 h).

Outcome (continuous)	Sleep duration				
	Insufficient (< 7 h)		Normal (7-8 h)	Excessive (> 8 h)	
	β (95% CI)	*p* value		β (95% CI)	*p* value
LDL	0.04 (−1.75, 1.83)	0.966	Reference	−0.62 (−3.89, 2.66)	0.713
Total cholesterol	−1.31 (−3.44, 0.82)	0.230		−2.04 (−5.59, 1.51)	0.262
Triglycerides	−0.61 (−4.05, 2.83)	0.727		−3.01 (−7.11, 1.09)	0.153
TG/HDL ratio	0.01 (−0.09, 0.11)	0.871		−0.04 (−0.17, 0.09)	0.536
NHHR	0.05 (−0.01, 0.11)	0.111		0.01 (−0.08, 0.11)	0.817
VAI	0.01 (−0.06, 0.08)	0.839		−0.02 (−0.12, 0.07)	0.616

*Note:* Exposure is categorical: insufficient (< 7 h), normal (7-8 h, reference), excessive (> 8 h). Outcomes are continuous indices (units shown in row labels). Values are adjusted differences in means (β) with 95% CIs from survey-weighted linear regression. Models adjust for sex, age, race/ethnicity, education, marital status, poverty-income ratio, BMI, diabetes, hypertension, smoking, and alcohol use. *p* values are from Wald tests; significant values (*p* < 0.05) are bolded. Units: LDL, total cholesterol, triglycerides in mg/dL; TG/HDL ratio, NHHR (non-HDL-C/HDL), and VAI are dimensionless.

**Table 5:** Survey-weighted multivariable linear regression of lipid and anthropometric indices by sleep disturbance status (reference = absent), Model III.

**Outcome (continuous)**	**Sleep disturbance**		
	**Absent**	**Present**	
		**β (95% CI)**	**p** **value**

HDL (mg/dL)	Reference	0.55 (−0.48, 1.58)	0.2965
LDL (mg/dL)		−0.77 (−2.94, 1.40)	0.4889
Total cholesterol (mg/dL)		0.63 (−1.74, 2.99)	0.6042
Triglycerides (mg/dL)		4.20 (−0.03, 8.42)	0.0544
TG/HDL ratio		0.09 (−0.03, 0.21)	0.1607
NHHR		−0.01 (−0.08, 0.05)	0.7120
TyG		0.04 (−0.00, 0.07)	0.0678
TyG–WHtR		**0.05 (0.02, 0.08)**	**0.0044**
TyG–WC		**0.09 (0.03, 0.15)**	**0.0033**
TyG–BMI		1.11 (−0.01, 2.23)	0.0543
VAI		0.06 (−0.03, 0.15)	0.1800
LAP		**2.62 (0.51, 4.73)**	**0.0165**
CI		**0.012 (0.001, 0.011)**	**0.0087**
BRI		**0.070 (0.010, 0.123)**	**0.0141**
ABSI		**0.001 (0.001, 0.012)**	**0.0069**
WWI		**0.001 (0.001, 0.002)**	**0.0170**

*Note:* Exposure is binary (present vs. absent, reference). Outcomes are continuous indices (units in row labels). Values are adjusted differences in means (β) with 95% confidence intervals from survey-weighted linear regression (Model III). Models adjust for sex, age, race/ethnicity, education, marital status, poverty-income ratio, BMI, diabetes, hypertension, smoking, and alcohol use. Significant results (*p* < 0.05 and 95% CI excluding 0) are bolded. Units: LDL-C, total cholesterol, triglycerides in mg/dL; TG/HDL ratio, NHHR (non-HDL-C/HDL), TyG-derived metrics, VAI, LAP, CI, BRI, ABSI, and WWI are dimensionless.

## Data Availability

Datasets used in this study can be found in online repositories (https://www.cdc.gov/nchs/nhanes/index.htm).

## Supporting Information

Table S1: Threshold effect analysis of sleep duration on nonlinear lipid and anthropometric index using a two-piecewise linear regression model in the NHANES 2005–2020 prepandemic. Significant *p* values are bolded.

Table S2: Multivariate linear analysis of association between sleep duration groups and nonlinear novel lipid and anthropometric indices, weighted.

Table S3: Multivariate linear analysis of association between sleep disturbance and novel lipid and anthropometric indices, weighted.

Figures S1–S16: Plots of crude estimated smoothing spine function of sleep duration with 95% CI band for generalized additive model (GAM) when the response variables are HDL (mg/dL), LDL (mg/dL), total cholesterol (mg/dL), TGs (mg/dL), TG/HDL, NHHR, TyG, TyG–WHtR, TyG–WC, TyG–BMI, VAI, LAP, CI, ABSI, BRI, and WWI.
