# Supplementary material for: Association Between Novel Lipid and Anthropometric Indices and Sleep Duration and Disturbance: A Cross-Sectional NHANES Study 2005–2020
Source: Int J Endocrinol. 2025 Oct 30;2025:9976711. doi: 10.1155/ije/9976711 (PMC12591825; doi:10.1155/ije/9976711)
Supplement: Supporting Information — Additional supporting information can be found online in the Supporting Information section. [file 9976711.f1.docx]

**Supplementary material**

Table S1: Threshold effect analysis of sleep duration on non-linear lipid and anthropometric index using a two-piecewise linear regression model in the NHANES 2005-2020 pre-pandemic. Significant p values are bolded

Table S2: Multivariate linear analysis of association between sleep duration groups and non-linear novel lipid and anthropometric indices, weighted.

Table S3: Multivariate linear analysis of association between sleep disturbance and novel lipid and anthropometric indices, weighted.

Figure S1: Plots of crude estimated smoothing spline function of sleep duration with 95% CI band for generalised additive model (GAM) when the response variable is HDL (mg/dL)

Figure S2: Plots of crude estimated smoothing spline function of sleep duration with 95% CI band for generalised additive model (GAM) when the response variable is LDL (mg/dL)

Figure S3: Plots of crude estimated smoothing spline function of sleep duration with 95% CI band for generalised additive model (GAM) when the response variable is Total cholesterol (mg/dL)

Figure S4: Plots of crude estimated smoothing spline function of sleep duration with 95% CI band for generalised additive model (GAM) when the response variable is Triglycerides (mg/dL)

Figure S5: Plots of crude estimated smoothing spline function of sleep duration with 95% CI band for generalised additive model (GAM) when the response variable is TG/HDL

Figure S6: Plots of crude estimated smoothing spline function of sleep duration with 95% CI band for generalised additive model (GAM) when the response variable is NHHR

Figure S7: Plots of crude estimated smoothing spline function of sleep duration with 95% CI band for generalised additive model (GAM) when the response variable is TyG

Figure S8: Plots of crude estimated smoothing spline function of sleep duration with 95% CI band for generalised additive model (GAM) when the response variable is TyG-WhTR

Figure S9: Plots of crude estimated smoothing spline function of sleep duration with 95% CI band for generalised additive model (GAM) when the response variable is TyG-WC

Figure S10: Plots of crude estimated smoothing spline function of sleep duration with 95% CI band for generalised additive model (GAM) when the response variable is TyG-BMI

Figure S11: Plots of crude estimated smoothing spline function of sleep duration with 95% CI band for generalised additive model (GAM) when the response variable is VAI

Figure S12: Plots of crude estimated smoothing spline function of sleep duration with 95% CI band for generalised additive model (GAM) when the response variable is LAP

Figure S13: Plots of crude estimated smoothing spline function of sleep duration with 95% CI band for generalised additive model (GAM) when the response variable is CI

Figure S14: Plots of crude estimated smoothing spline function of sleep duration with 95% CI band for generalised additive model (GAM) when the response variable is ABSI

Figure S15: Plots of crude estimated smoothing spline function of sleep duration with 95% CI band for generalised additive model (GAM) when the response variable is BRI

Figure S16: Plots of crude estimated smoothing spline function of sleep duration with 95% CI band for generalised additive model (GAM) when the response variable is WWI

| **Table S1** Threshold effect analysis of sleep duration on non-linear lipid and anthropometric index using a two-piecewise linear regression model in the NHANES 2005-2020 pre-pandemic. Significant p values are bolded | | | | | |
| --- | --- | --- | --- | --- | --- |
| Threshold effect analysis | *Adjusted β (95% CI) | *p*-value | Threshold effect analysis | *Adjusted β (95% CI) | *p*-value |
| HDL (mg/dL) | | | BRI | | |
| Linear regression | 0.156 (-0.032, 0.344) | 0.104 | Linear regression | 0.025 (0.015, 0.034) | **<0.001** |
| Two-piecewise linear regression | |  | Two-piecewise linear regression | |  |
| Inflection points (K1,K2) | 5.5, 7.5 |  | Inflection points (K1,K2) | 4.5, 7.5 |  |
| <5.5 | -0.440 (-1.231, 0.351) | 0.276 | <4.5 | 0.027 (-0.046, 0.099) | 0.469 |
| 5.5-7.5 | 0.824 (0.201, 1.447) | **0.010** | 4.5-7.5 | -0.014 (-0.037, 0.009) | 0.225 |
| >7.5 | 0.016 (-0.517, 0.548) | 0.944 | >7.5 | 0.082 (0.054, 0.109) | **<0.001** |
| Log-likelihood ratio test | **0.025** |  | Log-likelihood ratio test | **<0.001** |  |
| TyG-WHtR | | | TyG-WC | | |
| Linear regression | 0.003 (-0.003, 0.009) | 0.3867 | Linear regression | 0.000 (-0.010, 0.011) | 0.964 |
| Two-piecewise linear regression | |  | Two-piecewise linear regression | |  |
| Inflection points (K) | 6.5 |  | Inflection points (K) | 6.5 |  |
| <6.5 | -0.021 (-0.035, -0.007) | **0.003** | <6.5 | -0.032 (-0.057, -0.008) | **0.010** |
| >6.5 | 0.0170 (0.007, 0.026) | **<0.001** | >6.5 | 0.019 (0.003, 0.036) | **0.023** |
| Log-likelihood ratio test | **<0.001** |  | Log-likelihood ratio test | **0.004** |  |
| TyG-BMI | | | TyG | | |
| Linear regression | -0.179 (-0.399, 0.039) | 0.107 | Linear regression | -0.008 (-0.015, -0.001) | **0.039** |
| Two-piecewise linear regression | |  | Two-piecewise linear regression | |  |
| Inflection points (K) | 6.5 |  | Inflection points (K) | 5.5 |  |
| <6.5 | -0.617 (-1.134, -0.010) | **0.019** | <5.5 | -0.023 (-0.052, 0.005) | 0.100 |
| >6.5 | 0.080 (-0.273, 0.434) | 0.656 | >5.5 | -0.004 (-0.0136, 0.0051) | 0.368 |
| Log-likelihood ratio test | 0.067 |  | Log-likelihood ratio test | 0.25 |  |
| CI | | | ABSI | | |
| Linear regression | 0.001 (0.001, 0.002) | **0.002** | Linear regression | 0.0001 (0.0000, 0.0001) | **0.001** |
| Two-piecewise linear regression | |  | Two-piecewise linear regression | |  |
| Inflection points (K1,K2) | 4.5, 7.5 |  | Inflection points (K1,K2) | 4.5, 7.5 |  |
| <4.5 | 0.003 (-0.004, 0.009) | 0.374 | <4.5 | 0.0002 (-0.0002, 0.0006) | 0.352 |
| 4.5-7.5 | -0.002 (-0.004, 0.000) | 0.095 | 4.5-7.5 | -0.0001 (-0.0002, 0.0000) | 0.062 |
| >7.5 | 0.006 (0.004, 0.008) | **<0.001** | >7.5 | 0.0004 (0.0002, 0.0005) | **<0.001** |
| Log-likelihood ratio test | **<0.001** |  | Log-likelihood ratio test | **<0.001** |  |
| LAP | | | WWI | | |
| Linear regression | -0.179 (-0.591, 0.232) | 0.394 | Linear regression | 0.0134 (0.0061, 0.0208) | **0.0004** |
| Two-piecewise linear regression | |  | Two-piecewise linear regression | |  |
| Inflection points (K) | 6.5 |  | Inflection points (K1,K2) | 4, 6.5 |  |
| <6.5 | -1.326 (-2.301, -0.351) | **0.008** | <4 | 0.0003 (-0.0003, 0.0009) | 0.271 |
| >6.5 | 0.502 (-0.165,1.169) | 0.140 | 4-6.5 | -0.0003 (-0.0006, 0.0001) | 0.147 |
| Log-likelihood ratio test | **0.011** |  | >6.5 | 0.0004 (0.0003, 0.0006) | **<0.001** |
|  | | | Log-likelihood ratio test | **<0.001** |  |
| *Adjusted for Gender, Age, Educational level, Race, Marital status, PIR, BMI, Diabetes, Hypertension, Smoking status, Alcohol use.  HDL: High-density lipoprotein; TyG: Triglyceride-glucose index; TyG-WHtR: TyG related to WHtR; TyG-WC: TyG related to WC; TyG-BMI: TyG related to BMI; LAP: Lipid accumulation product; CI: Conicity index; BRI: Body roundness index; ABSI: a body shape index, WWI: Weight-adjusted waist index. | | | | | |

| **Table S2** Multivariate linear analysis of association between sleep duration groups and non-linear novel lipid and anthropometric indices, weighted. | | | | | | | | | | | |
| --- | --- | --- | --- | --- | --- | --- | --- | --- | --- | --- | --- |
|  |  | **LDL (mg/dL)** | | | **Total Cholesterol (mg/dL)** | | | **Triglycerides (mg/dL)** | | |  |
|  |  | Model I | Model II | Model III | Model I | Model II | Model III | Model I | Model II | Model III |  |
| **Sleep duration** | Insufficient (<7h) | 0.12 (-1.75, 1.98) 0.9015 | 0.57 (-1.26, 2.39) 0.5430 | 0.04 (-1.75, 1.83) 0.9656 | -2.31 (-4.47, -0.14) 0.0393 | -0.90 (-3.06, 1.25) 0.4134 | -1.31 (-3.44, 0.82) 0.2295 | 2.02 (-1.51, 5.55) 0.2649 | 3.07 (-0.55, 6.68) 0.0991 | -0.61 (-4.05, 2.83) 0.7274 |  |
|  | Normal (7-8h) | Reference | | | | | | | | |  |
|  | Excessive (>8h) | -1.83 (-5.22, 1.56) 0.2930 | -1.14 (-4.56, 2.27) 0.5129 | -0.62 (-3.89, 2.66) 0.7133 | -3.06 (-6.79, 0.66) 0.1096 | -2.58 (-6.31, 1.15) 0.1775 | -2.04 (-5.59, 1.51) 0.2623 | -3.53 (-7.76, 0.71) 0.1058 | -1.62 (-5.77, 2.53) 0.4455 | -3.01 (-7.11, 1.09) 0.1531 |  |
|  |  | **TG/HDL Ratio** | | | **NHHR** | | | **VAI** | | |  |
|  |  | Model I | Model II | Model III | Model I | Model II | Model III | Model I | Model II | Model III |  |
| **Sleep duration** | Insufficient (<7h) | **0.13 (0.03, 0.23) 0.0150** | **0.12 (0.02, 0.23) 0.0225** | 0.01 (-0.09, 0.11) 0.8706 | **0.14 (0.07, 0.21) 0.0001** | **0.12 (0.05, 0.18) 0.0005** | 0.05 (-0.01, 0.11) 0.1105 | 0.07 (-0.00, 0.15) 0.0614 | **0.22 (0.05, 0.40) 0.0132** | 0.01 (-0.06, 0.08) 0.8391 |  |
|  | Normal (7-8h) | Reference | | | | | | | | |  |
|  | Excessive (>8h) | -0.07 (-0.21, 0.07) 0.3491 | 0.00 (-0.13, 0.14) 0.9705 | -0.04 (-0.17, 0.09) 0.5359 | -0.03 (-0.13, 0.08) 0.5812 | 0.02 (-0.08, 0.12) 0.7020 | 0.01 (-0.08, 0.11) 0.8166 | 0.03 (-0.06, 0.13) 0.5110 | 0.04 (-0.19, 0.26) 0.7375 | -0.02 (-0.12, 0.07) 0.6157 |  |
| All results presented as β (95% CI) p-value. Model I: crude; Model II: adjusted for Age, Gender, Educational level, Race, Marriage and PIR; Model III: adjusted for Age, Gender, Educational level, Race, Marriage, PIR, BMI, Diabetes status, Hypertension, Smoking status and Alcohol intake. Significant P values are bolded.  LDL: Low-density lipoprotein; TG/HDL ratio: Total cholesterol to high-density lipoprotein ratio; NHHR: non-high-density lipoprotein cholesterol to high-density lipoprotein cholesterol ratio; VAI: Visceral adiposity index | | | | | | | | | | |  |

| **Table S3** Multivariate linear analysis of association between sleep disturbance and novel lipid and anthropometric indices, weighted. | | | | | | | | | | | | | |
| --- | --- | --- | --- | --- | --- | --- | --- | --- | --- | --- | --- | --- | --- |
|  |  | **HDL (mg/dL)** | | | **LDL (mg/dL)** | | | **Total Cholesterol (mg/dL)** | | | **Triglycerides (mg/dL)** | | |
|  |  | Model I | Model II | Model III | Model I | Model II | Model III | Model I | Model II | Model III | Model I | Model II | Model III |
| **Sleep disturbance** | Absent | Reference | | | Reference | | | Reference | | | Reference | | |
|  | Present | 0.72 (-0.39, 1.84) 0.2054 | -0.88 (-1.97, 0.21) 0.1185 | 0.55 (-0.48, 1.58) 0.2965 | -0.11 (-2.31, 2.08) 0.9217 | -0.82 (-2.99, 1.35) 0.4612 | -0.77 (-2.94, 1.40) 0.4889 | 2.43 (-0.13, 4.98) 0.0651 | 0.10 (-2.31, 2.51) 0.9356 | 0.63 (-1.74, 2.99) 0.6042 | **9.05 (4.67, 13.42) 0.0001** | **8.95 (4.63, 13.27) 0.0001** | 4.20 (-0.03, 8.42) 0.0544 |
|  |  | **TG/HDL Ratio** | | | **NHHR** | | | **TyG** | | | **TyG-WHTR** | | |
|  |  | Model I | Model II | Model III | Model I | Model II | Model III | Model I | Model II | Model III | Model I | Model II | Model III |
| **Sleep disturbance** | Absent | Reference | | | Reference | | | Reference | | | Reference | | |
|  | Present | **0.18 (0.06, 0.30) 0.0041** | **0.25 (0.12, 0.37) 0.0002** | 0.09 (-0.03, 0.21) 0.1607 | 0.01 (-0.06, 0.08) 0.7733 | **0.07 (0.00, 0.14) 0.0451** | -0.01 (-0.08, 0.05) 0.7120 | **0.11 (0.07, 0.15) <0.0001** | **0.10 (0.06, 0.14) <0.0001** | 0.04 (-0.00, 0.07) 0.0678 | **0.32 (0.25, 0.38) <0.0001** | **0.27 (0.20, 0.33) <0.0001** | **0.05 (0.02, 0.08) 0.0044** |
|  |  | **TyG-WC** | | | **TyG-BMI** | | | **VAI** | | | **LAP** | | |
|  |  | Model I | Model II | Model III | Model I | Model II | Model III | Model I | Model II | Model III | Model I | Model II | Model III |
| **Sleep disturbance** | Absent | Reference | | | Reference | | | Reference | | | Reference | | |
|  | Present | **0.49 (0.37, 0.60) <0.0001** | **0.46 (0.35, 0.57) <0.0001** | **0.09 (0.03, 0.15) 0.0033** | **16.57 (12.13, 21.00) <0.0001** | **16.41 (12.03, 20.79) <0.0001** | 1.11 (-0.01, 2.23) 0.0543 | **0.22 (0.13, 0.31) <0.0001** | **0.19 (0.10, 0.28) 0.0001** | 0.06 (-0.03, 0.15) 0.1800 | **10.61 (7.82, 13.40) <0.0001** | **9.36 (6.65, 12.06) <0.0001** | **2.62 (0.51, 4.73) 0.0165** |
|  |  | **CI** | | | **BRI** | | | **ABSI** | | | **WWI** | | |
|  |  | Model I | Model II | Model III | Model I | Model II | Model III | Model I | Model II | Model III | Model I | Model II | Model III |
| **Sleep disturbance** | Absent | Reference | | | Reference | | | Reference | | | Reference | | |
|  | Present | **0.03 (0.02, 0.03) <0.0001** | **0.02 (0.01, 0.03) <0.0001** | **0.01 (0.00, 0.01) 0.0087** | **0.69 (0.54, 0.84) <0.0001** | **0.57 (0.42, 0.72) <0.0001** | **0.07 (0.01, 0.12) 0.0141** | **0.01 (0.01, 0.01) <0.0001** | **0.01 (0.00, 0.01) 0.0012** | **0.00 (0.00, 0.01) 0.0069** | **0.00 (0.00, 0.00) <0.0001** | **0.00 (0.00, 0.00) <0.0001** | **0.00 (0.00, 0.00) 0.0170** |
| All results presented as β (95% CI) p-value. Model I: crude; Model II: adjusted for Age, Gender, Educational level, Race, Marriage and PIR; Model III: adjusted for Age, Gender, Educational level, Race, Marriage, PIR, BMI, Diabetes status, Hypertension, Smoking status and Alcohol intake. Significant P values are bolded. | | | | | | | | | | | | | |

**
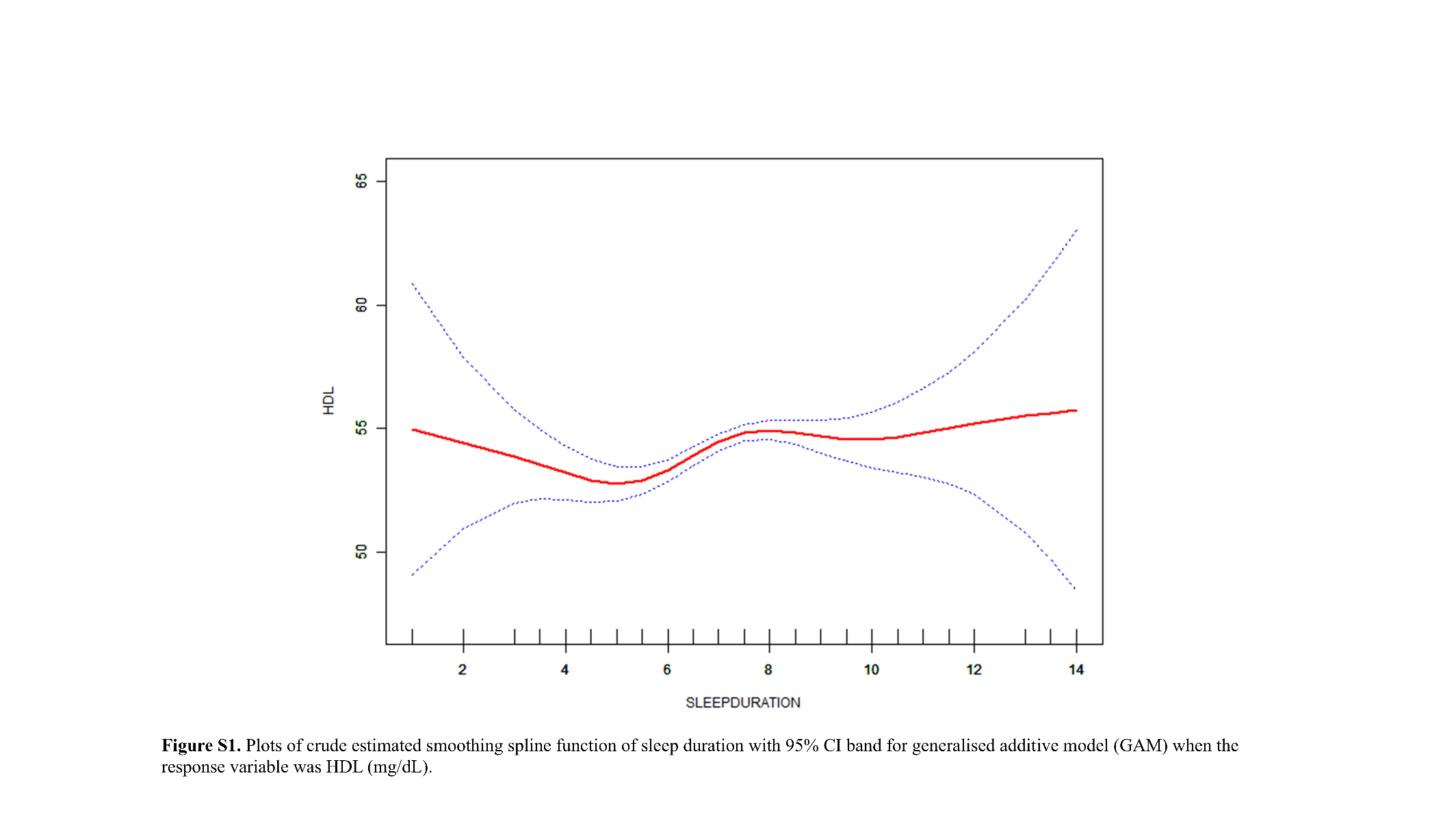
**

**
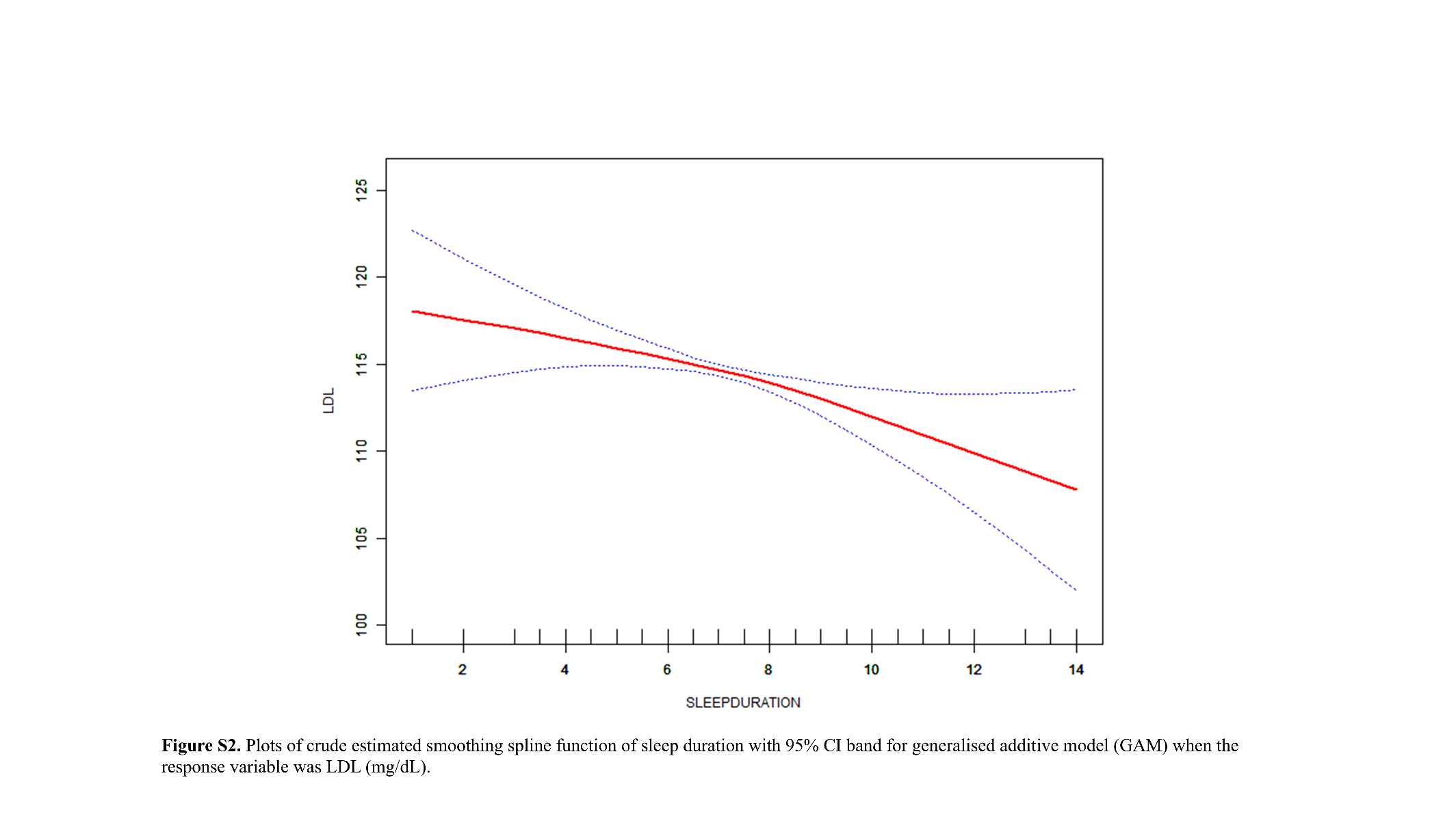
**

**
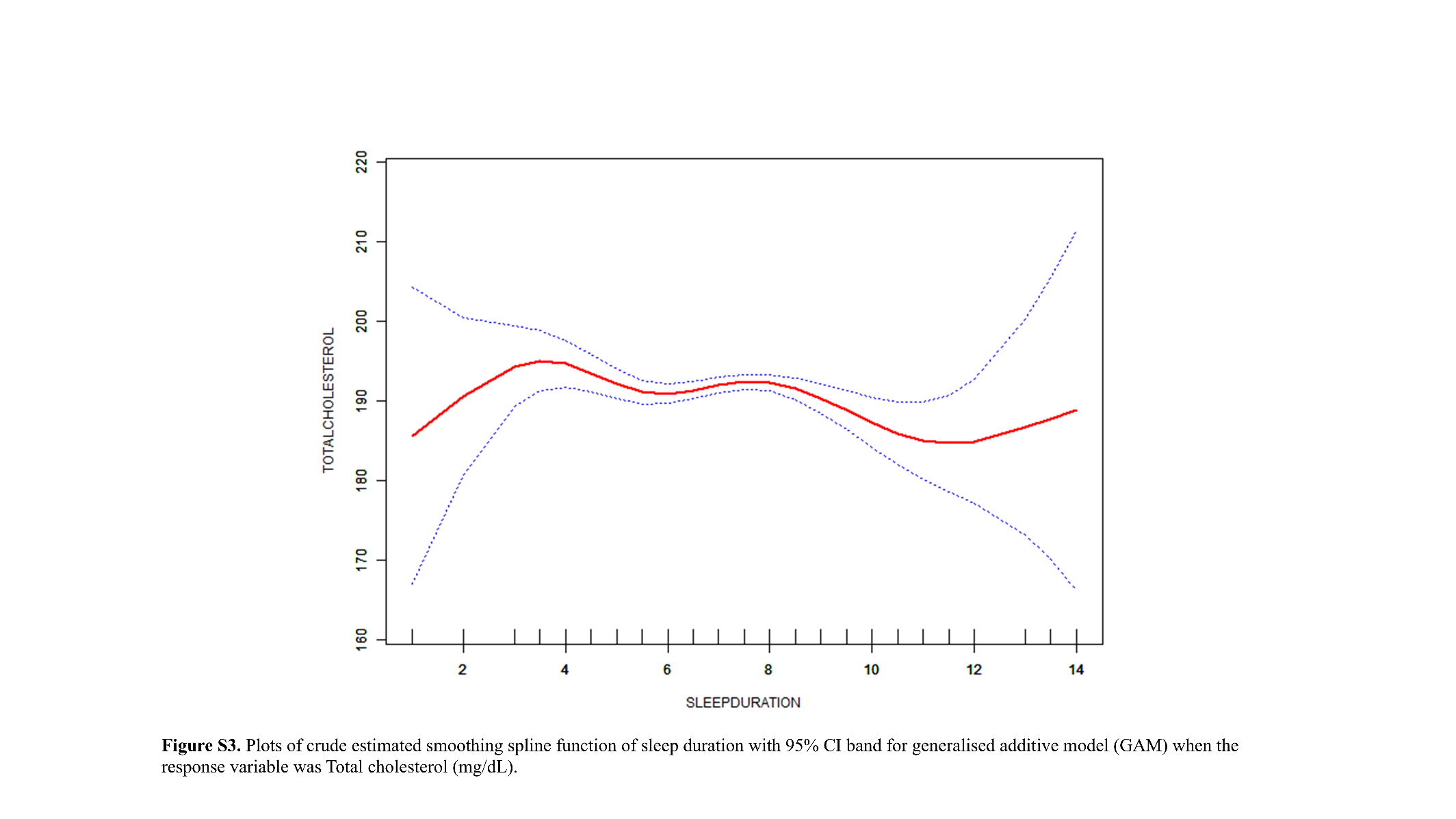
**

**
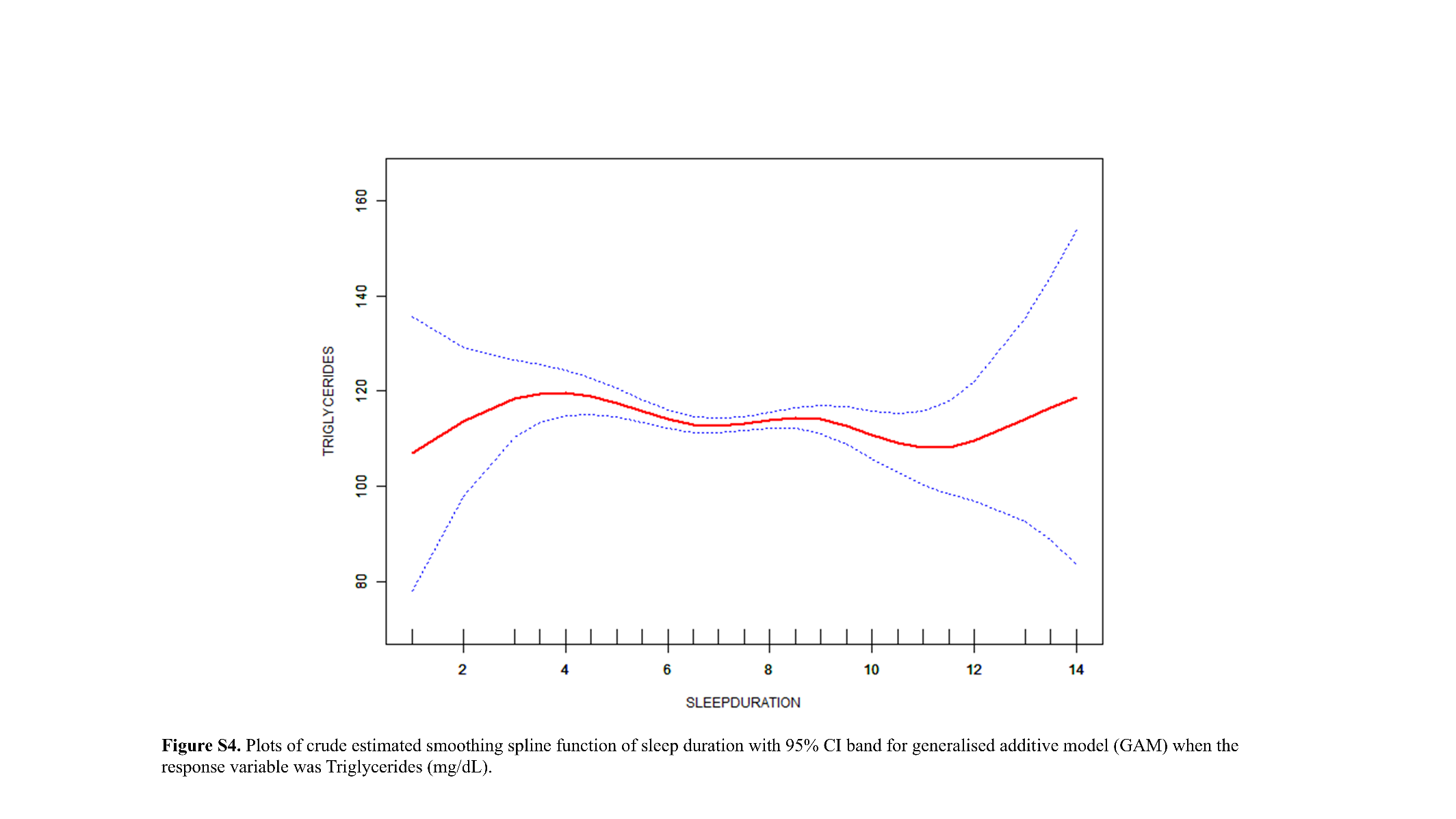
**

**
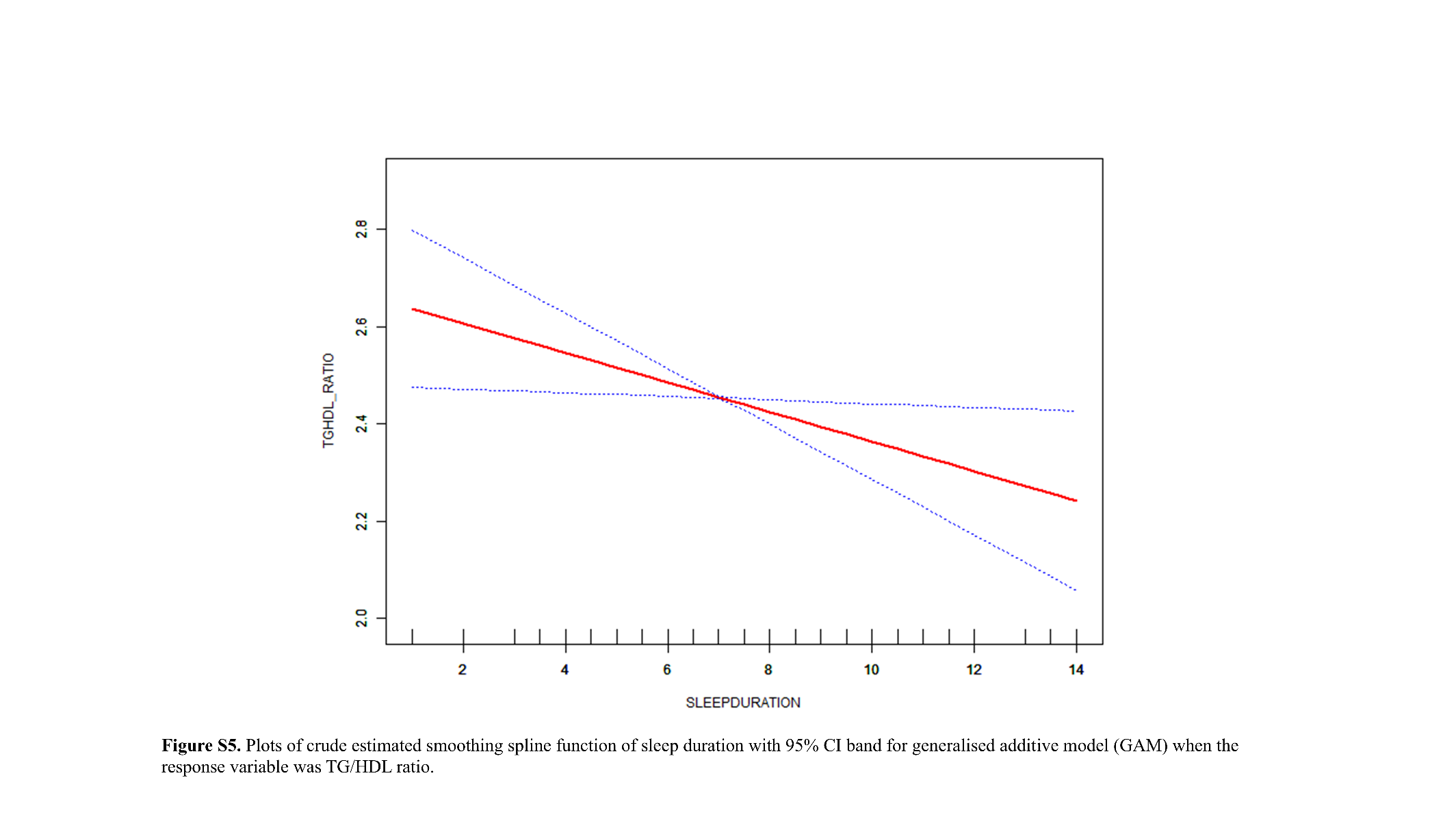
**

**
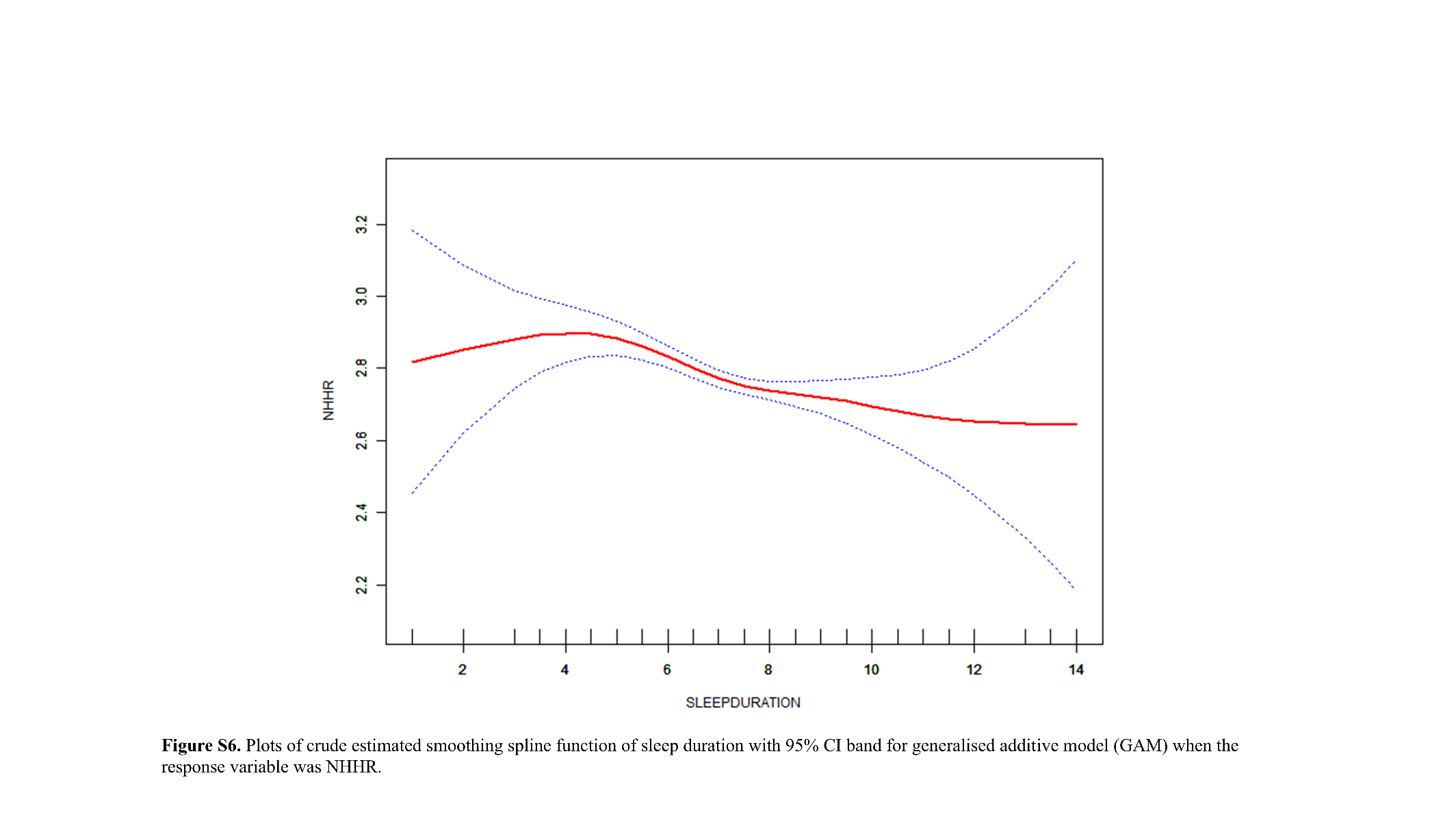
**

**
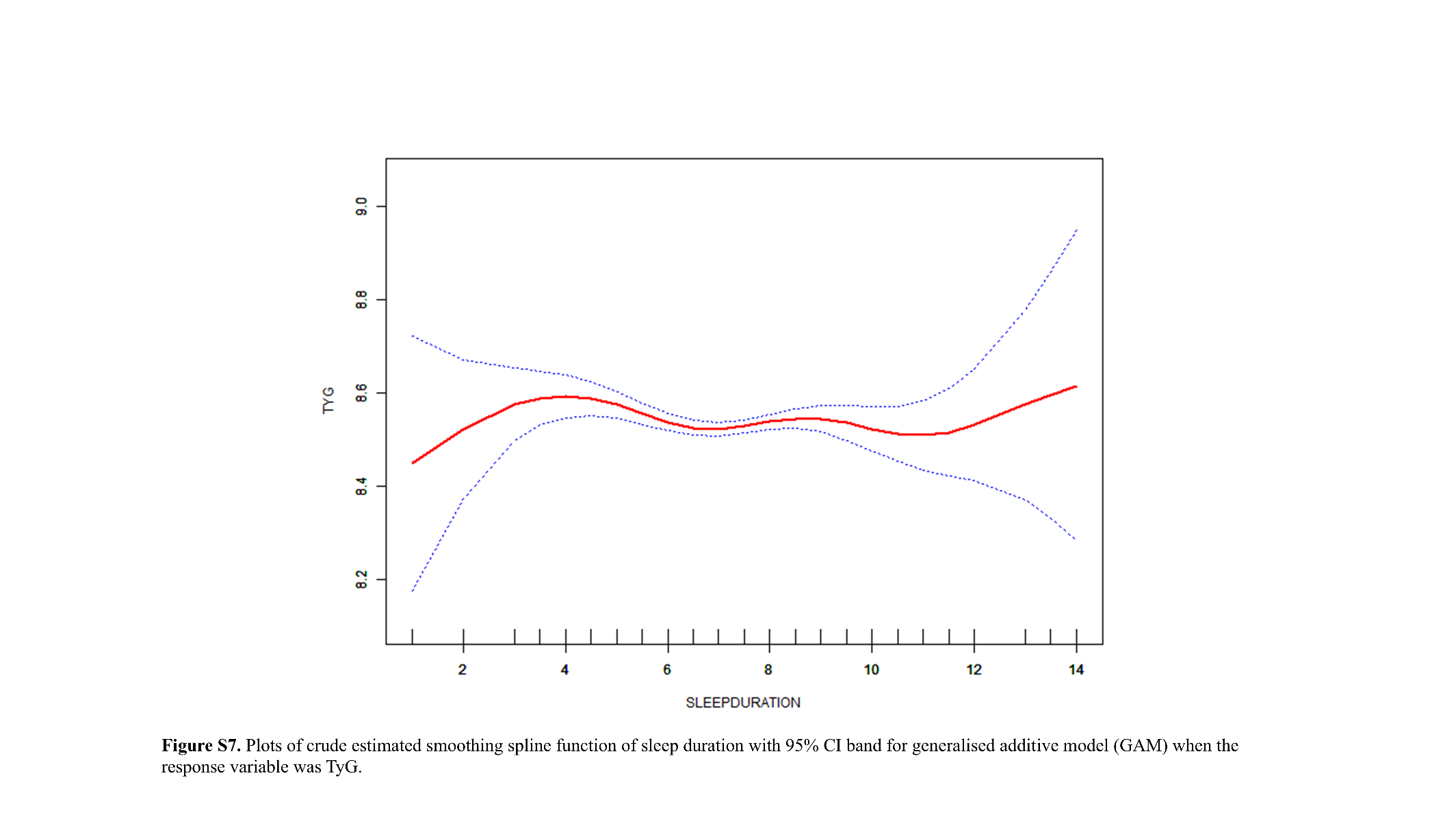
**

**
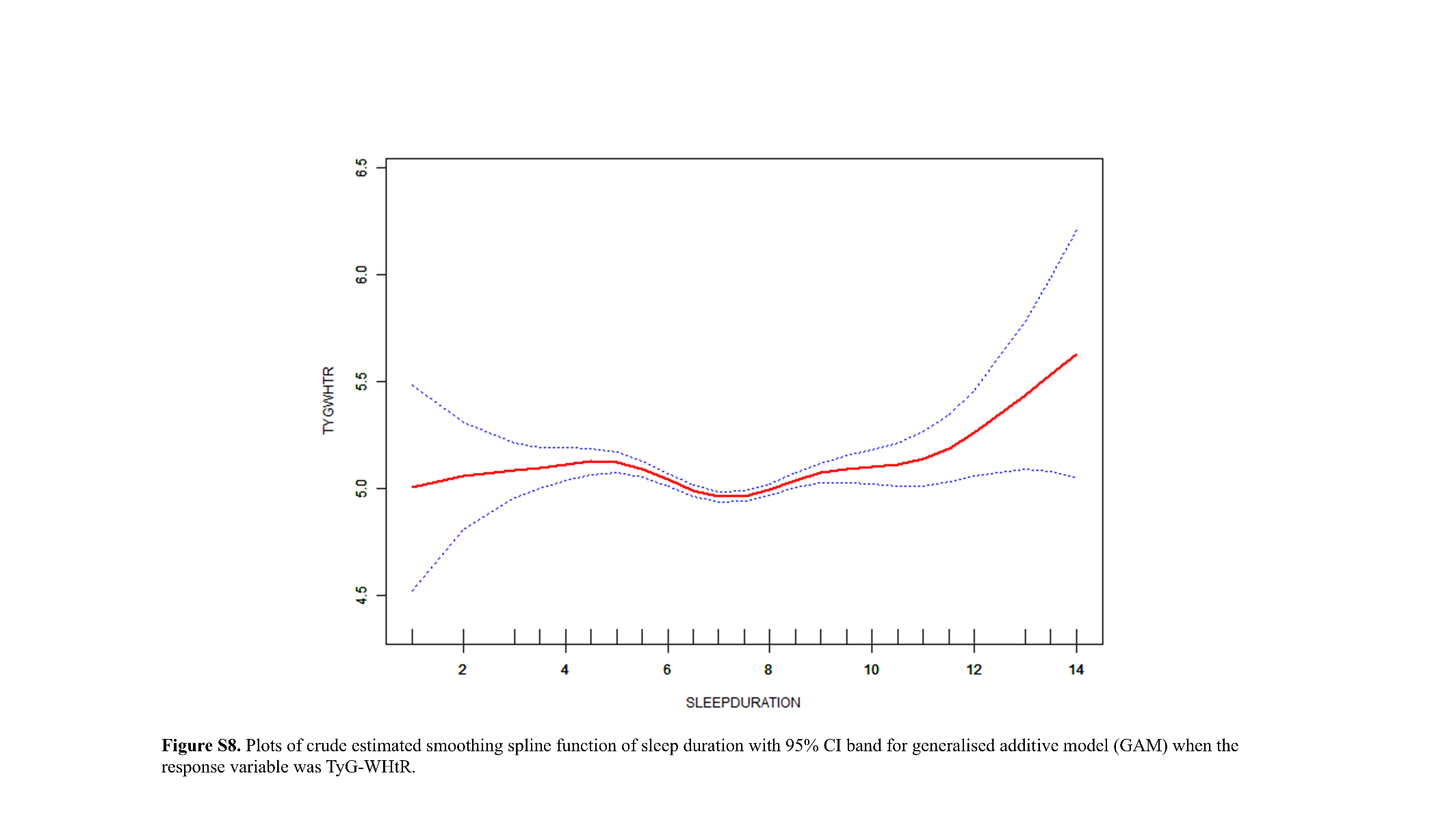
**

**
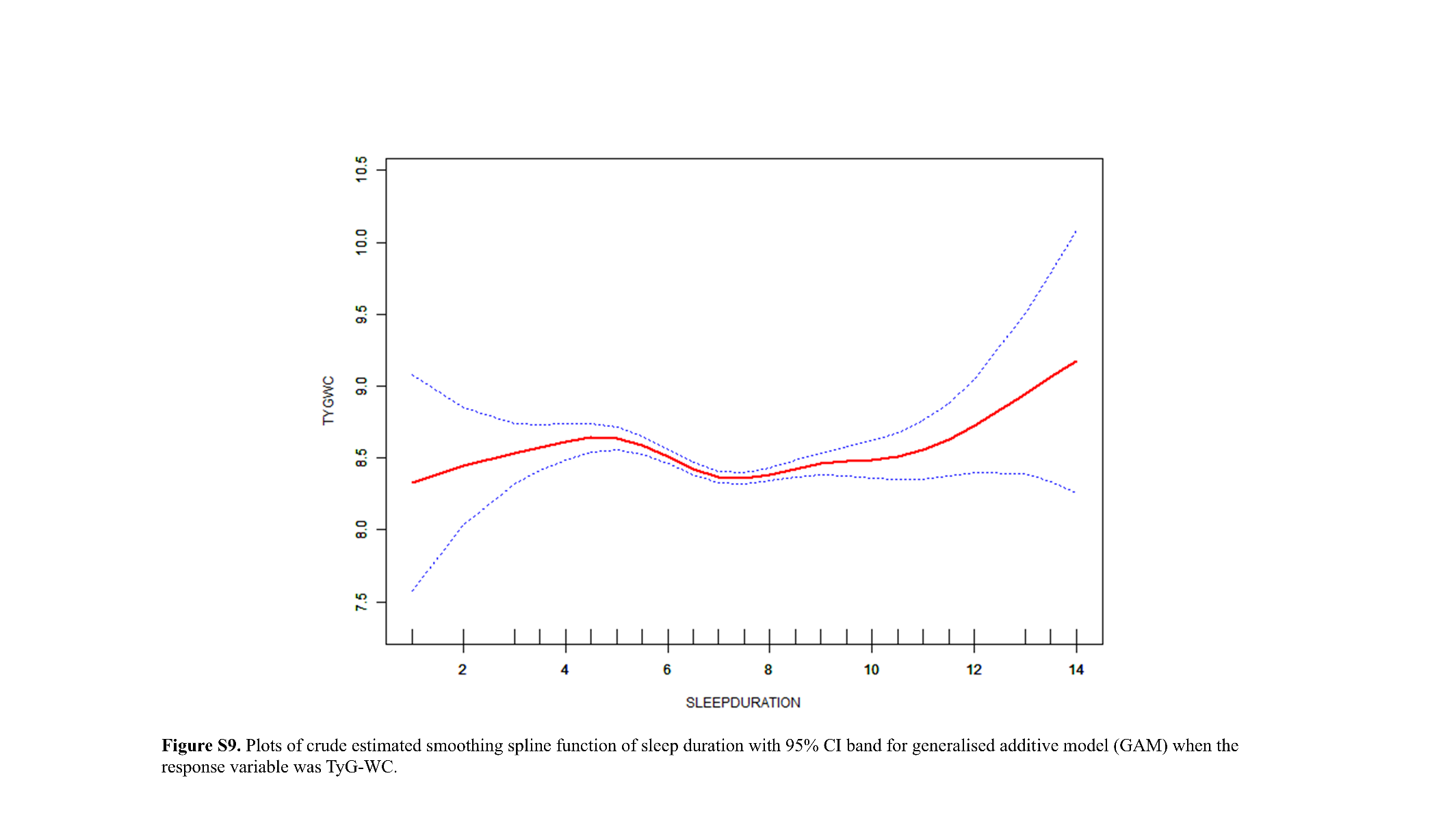
**

**
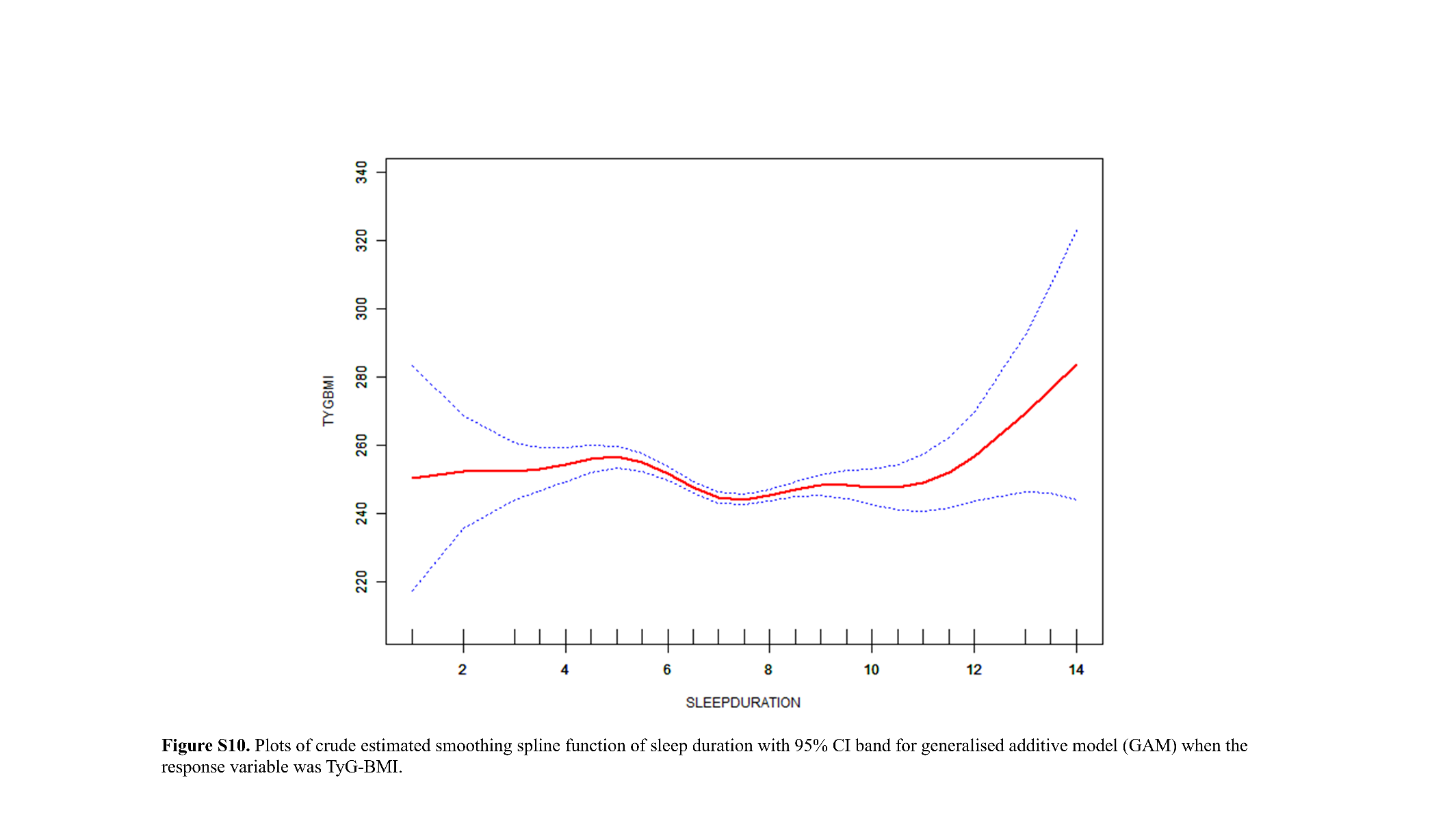
**

**
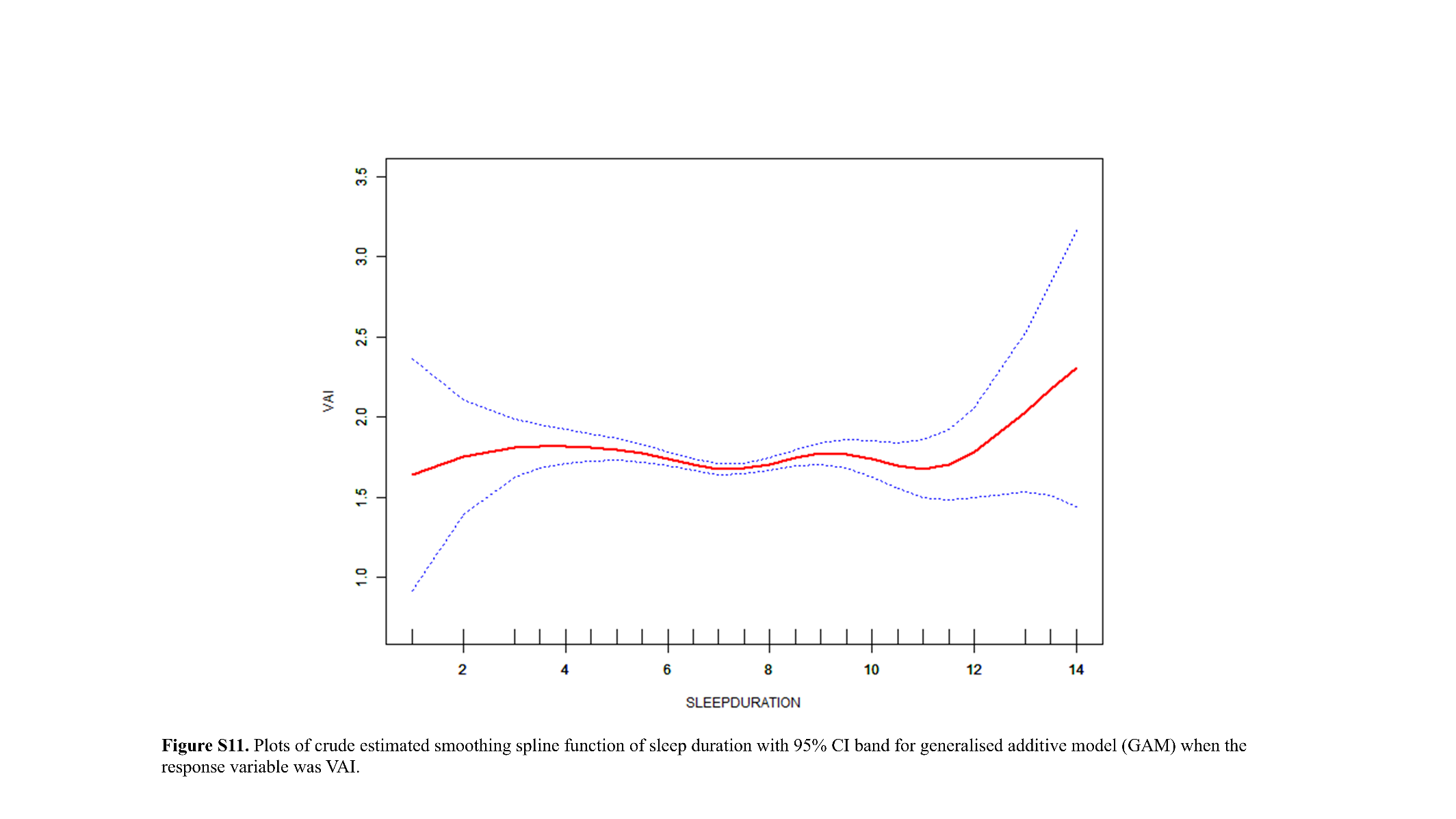
**

**
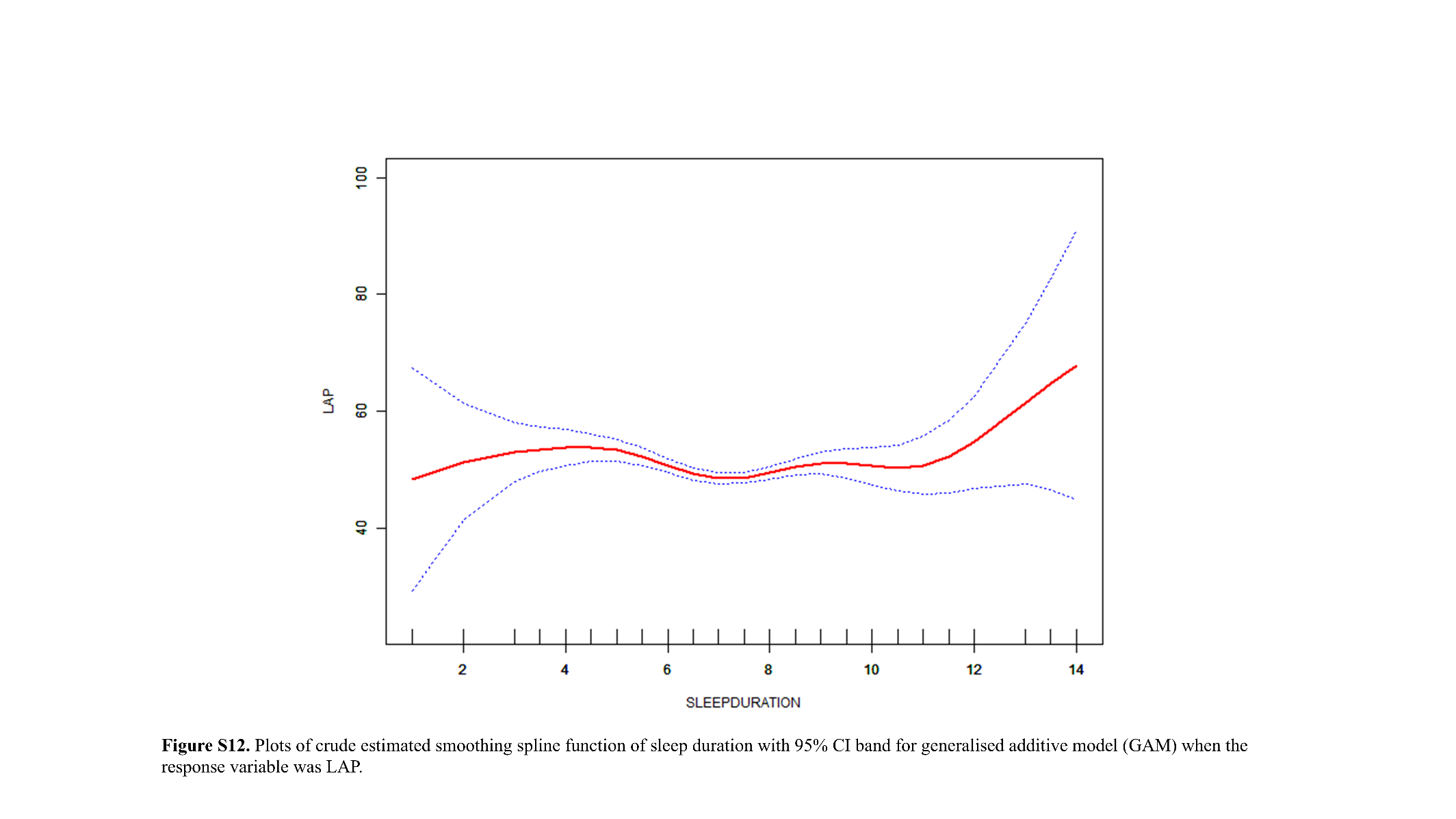
**

**
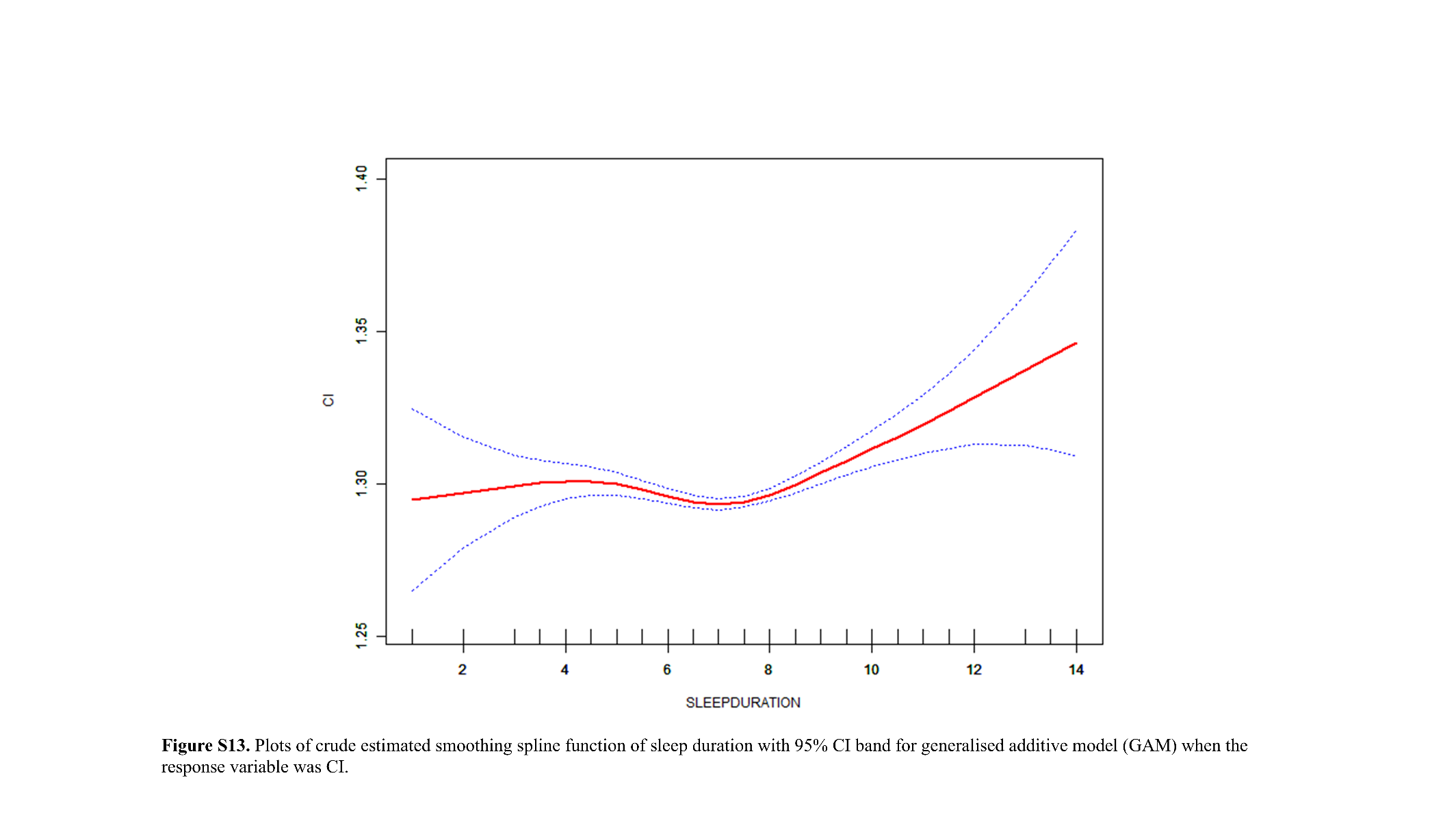
**

**
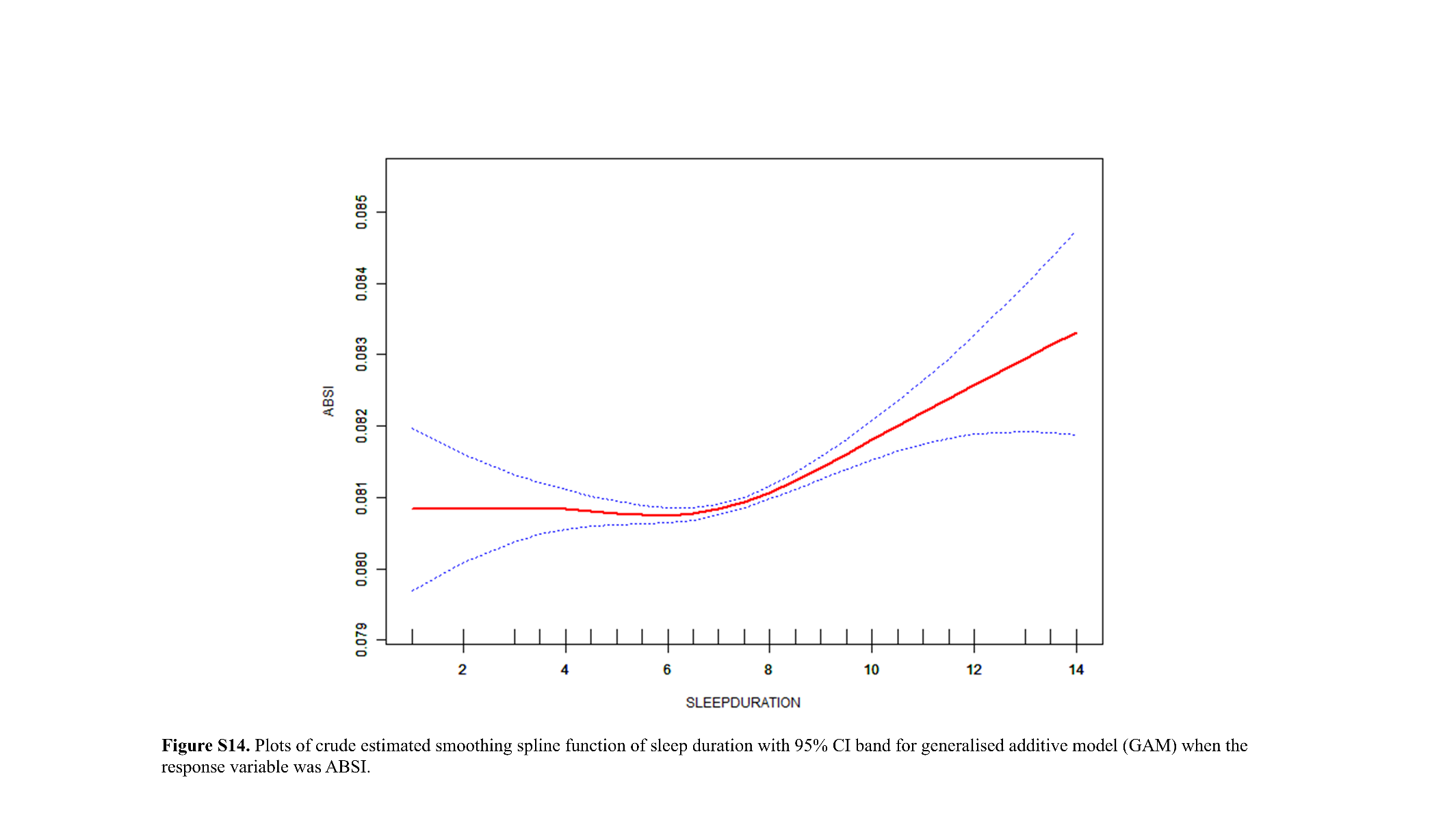
**

**
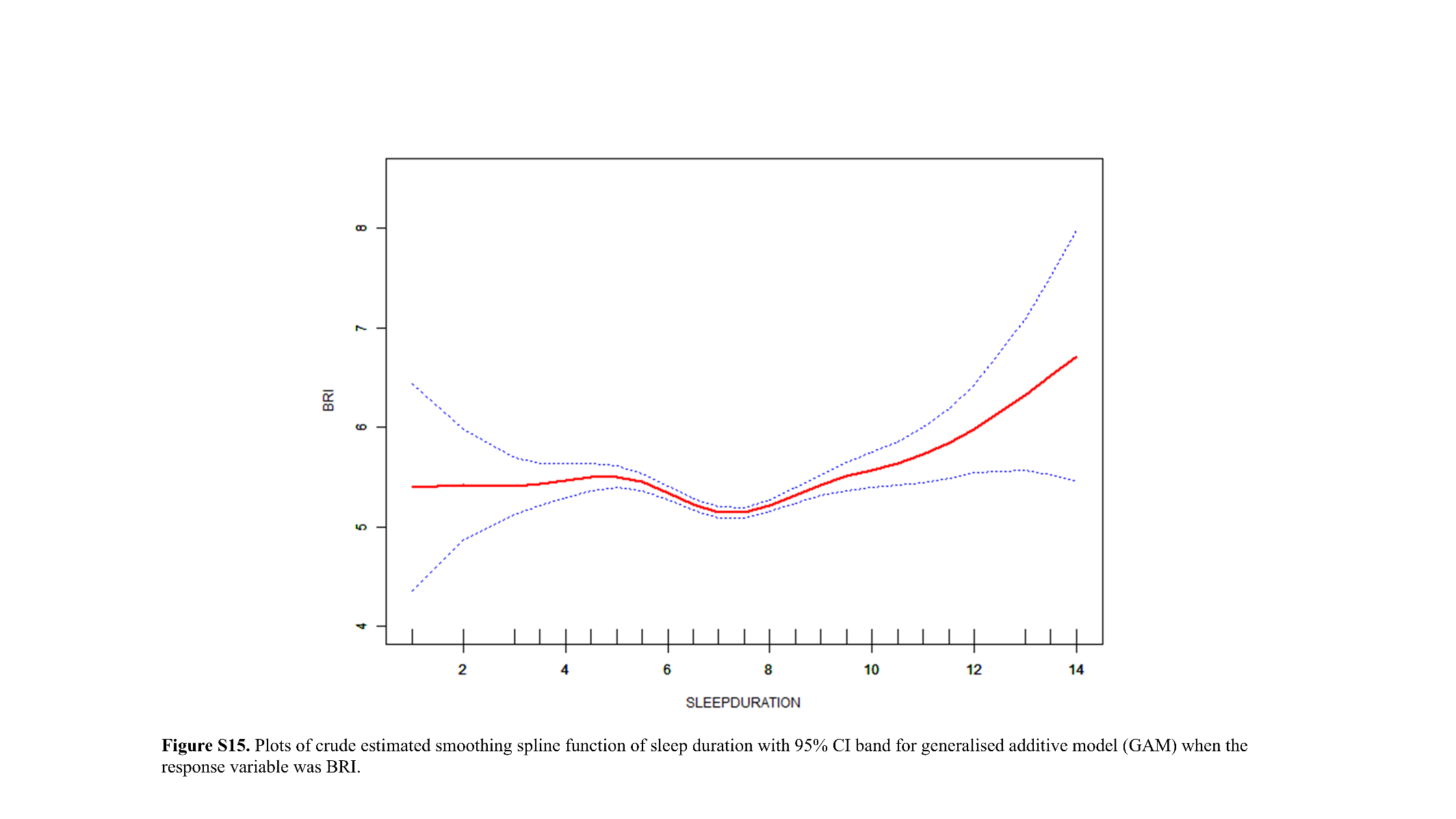
**

**
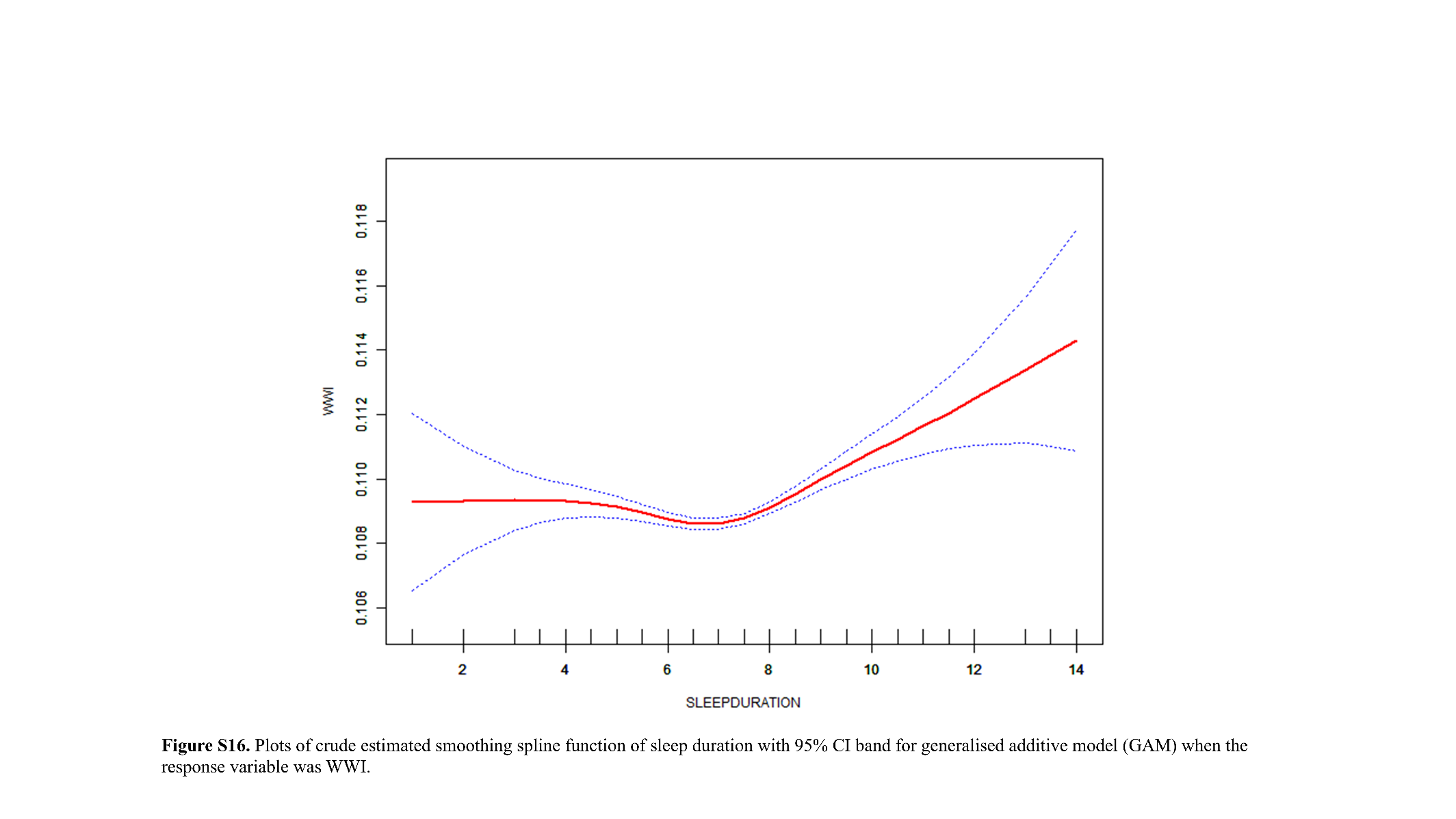
**
